# Supplementary material for: Trans‐generational epigenetic regulation associated with the amelioration of Duchenne Muscular Dystrophy
Source: EMBO Mol Med. 2020 Jun 29;12(8):e12063. doi: 10.15252/emmm.202012063 (PMC7411655; doi:10.15252/emmm.202012063)
Supplement: Supplementary file 1 — Expanded View Figures PDF [file EMMM-12-e12063-s001.pdf]

Expanded View Figures

Figure EV1. UPΔ44 iPSC characterization and genome editing strategy description.

A Representative phase contrast image of UPΔ44#3 pluripotent stem cell morphology. Scale bar 100 μm.

B qPCR analyses of pluripotency-associated genes (NANOG, OCT4, SOX2 and REX1) and of the direct inhibitor of OCT4 (NR2F2) expression in the patient-specific iPSC clone (UPΔ44#3) relative to the somatic cell control (UPΔ44 fibroblasts). PCRs were normalized against an internal control (GAPDH) and plotted relative to the expression level in the individual parent fibroblast cell lines set to a value of 1. The mean ± SEM of triplicates from one representative experiment is shown (n = 3).

C RT–PCR for the exogenous OCT4 mRNA in RNA extracted from UPΔ44 fibroblasts (UPΔ44 Fibro), fibroblasts collected 3 days after lentiviral infection with hSTEMCCA vector (Inf Fibro) and the patient-specific iPSC clone#3 (UPΔ44#3). GAPDH was used as control. Lane (−) indicates the negative control. Representative results are shown (n = 3).

D Normal karyotype of WT#1 and UPΔ44#3 iPSC clones (46, XY).

E Genetic identity with the parental cell line was assessed by PCR on gDNA. PCR was performed on gDNA from control and UPΔ44 fibroblasts cells and iPSCs amplifying the indicated DMD exons. Lane (−) indicates the negative control.

F Schematic representation of the gene-editing strategy. Vectors encoding Cas9 and sgRNA targeting the first exon of CELF2a gene, together with a donor plasmid containing a selection cassette flanked by Lox P sites and two homologous sequences (~800 bp each) to upstream and downstream regions of the first CELF2a exon, were co-transfected in UPΔ44 iPSCs. The edited clones were selected based on puromycin resistance, whose insertion was validated by PCR (F1-R1). Homozygous clones were selected based on the absence of the CELF2a genomic region assessed by RT–PCR using the indicated oligonucleotides (F2-R2).

G qPCR analysis of the indicated muscle differentiation markers in iPSCs prior (iPSC) and after the induction of muscle differentiation (Myo) in the indicated clones. Relative levels of mRNAs were calculated with the delta delta C_t method. qPCRs were normalized against an internal control (GAPDH) and plotted relative to the expression level in iPSCs (0) set to a value of 1. The mean ± SEM of triplicates from one representative experiment is shown (n = 3).

H Sanger sequencing data confirming the exon 45 skipping in the UPΔ44 CRISPR/Cas9 edited clones depleted for CELF2a (UPΔ44#3.1 and UPΔ44#3.5).

Source data are available online for this figure.

© 2020 The Authors

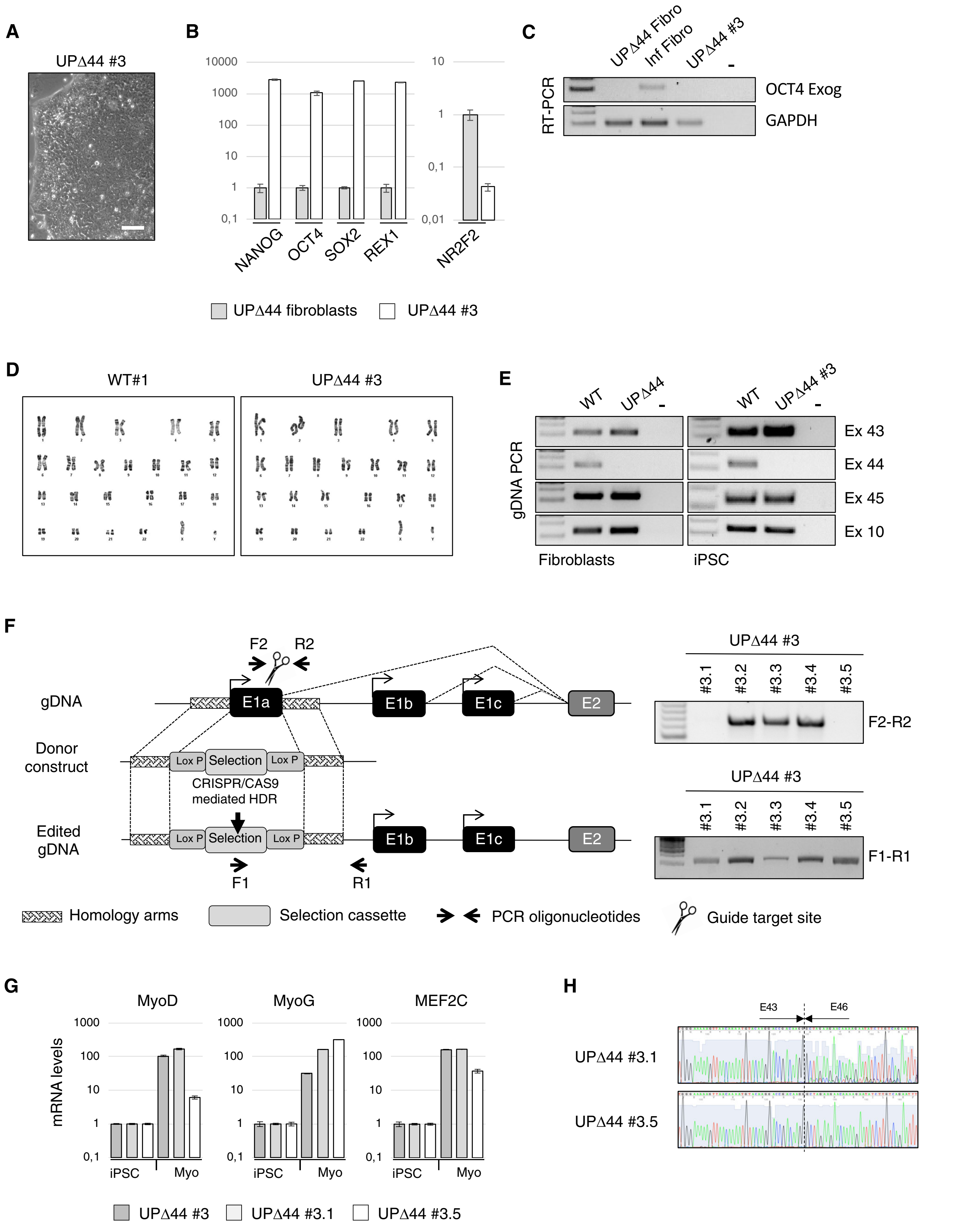

Figure EV1.

© 2020 The Authors

Figure EV2. GSΔ44 iPSC characterization and genome editing strategy description.

A Representative phase-contrast images of GSΔ44 (#2, #8) and GSM (#1) pluripotent stem cell clones' morphology. Scale bar 100 μm.
B qPCR analyses of pluripotency-associated genes (NANOG, OCT4, SOX2 and REX1) and of the direct inhibitor of OCT4 (NR2F2) in indicated patient-specific iPSC clones relative to the somatic cells control (fibroblasts). PCRs were normalized against an internal control (GAPDH) and plotted relative to the expression level in the individual parent fibroblast cell lines set to a value of 1. The mean ± SEM of triplicates from one representative experiment is shown (n = 3).
C RT–PCR for the exogenous OCT4 mRNA in RNA extracted from GSΔ44 fibroblasts (GSΔ44 Fibro), fibroblasts collected 3 days after lentiviral infection with hSTEMCCA vector (Inf Fibro), patient-specific iPSC clones #2 and 8 (GSΔ44#2 and GSΔ44#8) and the mother-specific iPSC clone #1 (GSM#1). GAPDH was used as control. Lane (−) indicates the negative control. Representative results are shown (n = 3).
D Normal karyotype of GSΔ44#2 and GSΔ44#8 iPSC clones (46, XY).
E Left panel: Schematic representation of GSΔ44-specific gDNA deletion on the DMD gene showing the location of the oligonucleotides used to amplify deletion boundaries. Middle and right panel: Genetic identity with the parental cell line was assessed by PCR on gDNA. PCR was performed on gDNA from control fibroblasts (middle panel) and iPSCs (right panel) amplifying the indicated DMD exons and the DMD deletion boundary characteristic of GSΔ44 and GSM individuals. Lane (−) indicates the negative control.
F qPCR analysis of the indicated muscle differentiation markers in iPSCs prior (iPSC) and after the induction of muscle differentiation (Myo) in the indicated clones. Relative levels of mRNAs were calculated with the delta delta C_t method. qPCRs were normalized against an internal control (GAPDH) and plotted relative to the expression level in iPSCs (iPSC) set to a value of 1. The mean ± SEM of triplicates from one representative experiment is shown (n = 3).
G RT–PCR analyses of CELF2a, MyoG, MyoD, Baf60c and Mef2c in control (WT1 and WT2) and in GSΔ44 myoblasts transfected with a plasmid for the overexpression of MyoD alone (epB-MyoD) or in combination with Baf60c (epB-Baf60c) collected before treatment (Un) or at the indicated time points. GAPDH was used as control. Representative results are shown (n = 3).
H Selection of GSΔ44-edited clones based on puromycin resistance, which was validated by PCR (F1-R1). Homozygous clones were selected based on the absence of the CELF2a genomic region assessed by RT–PCR using the indicated oligonucleotides (F2-R2) according to the schematic representation of the gene-editing strategy shown in (Fig EV1F).
I Sanger sequencing data confirming the exon 45 skipping in the GSΔ44#8.2 CRISPR/Cas9-edited clone depleted for CELF2a.

Source data are available online for this figure.

© 2020 The Authors

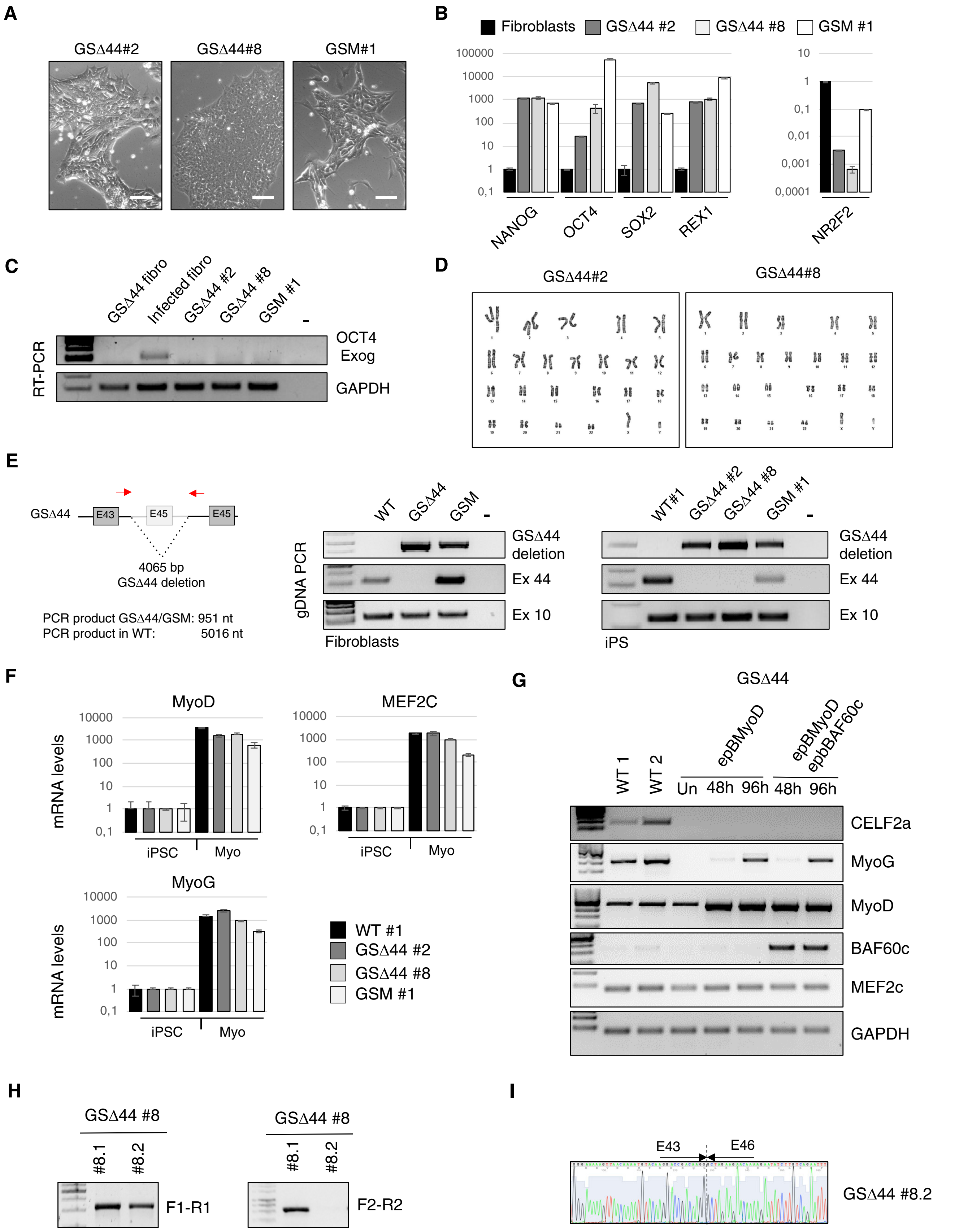

Figure EV2.

 © 2020 The Authors

Figure EV3. Analysis of CELF2a regulatory region.

A Scatterplots of ATAC-seq peak intensities between two conditions.
B ATAC-seq fragment sizes generated from WT and GSΔ44.
C RT–PCR analysis for CELF2a, CELF2b, CELF2c expression in WT and GSΔ44 myoblasts. GAPDH was used as the loading control. Lane (–) indicates the negative control. Representative results are shown (n = 3).
D DNA accessibility (ATAC-seq) and chromatin marks (ChIP-seq) signal in the first exon region of CELF2a and b isoforms using the UCSC genome browser. ATAC-seq peaks called in the two conditions are reported below each track. The differential peaks specific for one of the two conditions are reported in the tracks ATACseq_specific peaks_WT and ATACseq_specific peaks_GSΔ44. ChIP-seq signals for H3K4me3, H3K27ac and H3K27me3 in WT and GSΔ44 myoblasts are reported (WT myoblasts in light orange; GSΔ44-derived myoblasts in light blue). ChIP-seq for MyoD in control cells was obtained from ref. MacQuarrie *et al* (2013). Blue boxes highlight MyoD binding site peaks with differential DNA accessibility between WT final vs GSΔ44 (Peak 1 and 2).

© 2020 The Authors

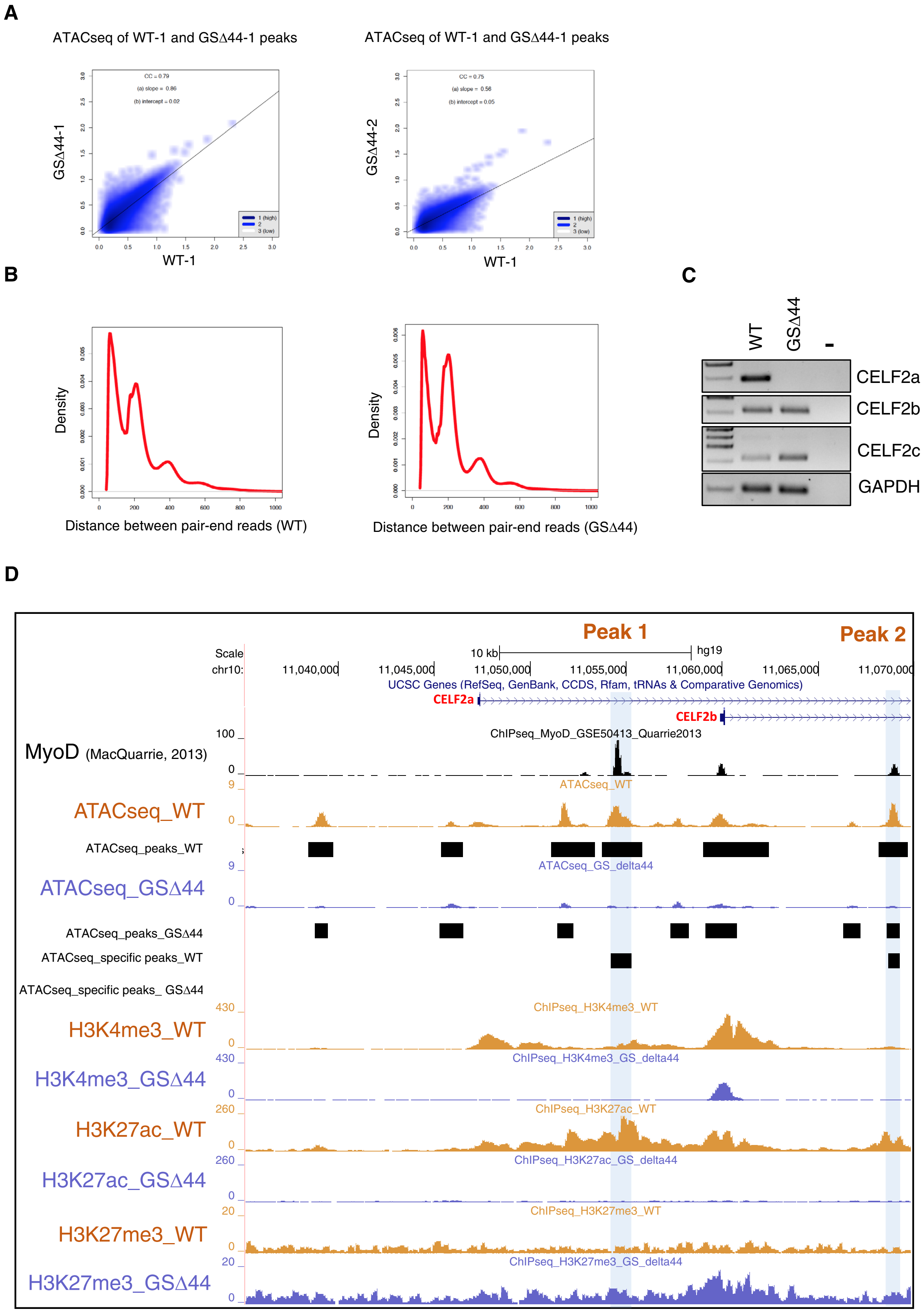

Figure EV3.

© 2020 The Authors

Figure EV4. Analysis of lncRNAs differentially expressed in GSΔ44.

A Screenshot of UCSC Genome Browser displaying annotated lncRNAs in the Celf2 locus (chr10 in GRCh37/hg19 assembly).
B RT–PCR for the indicated lncRNAs on extracts from WT, Δ44 and GSΔ44 myocytes. GAPDH was used as control. Representative results are shown (n = 3).
C RT–PCR of CELF2a and SFTA1P on cytoplasm (C) and nuclear (N) RNA obtained from GSΔ44 myoblasts transfected with a plasmid for SFTA1P overexpression (OE SFTA1P) or an empty vector (Empty) and incubated 48 h. GAPDH mRNA and pre-mRNA (pre-GAPDH) were used, respectively, as cytoplasm and nuclear controls. Lane (−) indicates the negative control. Representative results are shown (n = 3).
D RT–PCR for CELF2a and SFTA1P expression in RNA extracted from WT myoblasts treated with either a control gapmer (Gapmer SCR) or a gapmer against SFTA1P (Gapmer SFTA1P) and collected 2 days after transfection. GAPDH was used as control. Representative results are shown (n = 3).
E RT–PCR for SFTA1P and CELF2a expression in RNA extracted from GSΔ44 myoblasts transfected with an empty vector (Empty) or a plasmid for the overexpression of CELF2a (OE CELF2a) and collected after 48 h. GAPDH was used as control. Lane (−) indicates the negative control. Representative results are shown (n = 3).
F iPSCs' RNA analysed in Fig 2A was further analysed by RT–PCR for SFTA1P expression. GAPDH has been shown twice to make the image easier to understand. Representative results are shown (n = 3).
G Venn diagram showing the number of differentially expressed lncRNAs between WT and Δ44 (blue) and WT and GSΔ44 (orange) samples.
H RT–PCR on cytoplasm (C) nucleoplasm (N) and chromatin (Chr) GSΔ44 myoblast extracts showing the subcellular localization of DUXAP8. GAPDH mRNA and pre-mRNA (pre-GAPDH) were used as cytoplasm, nucleoplasm and chromatin controls. Lane (−) indicates the negative control. Representative results are shown (n = 3).
I RT–PCR on cytoplasm (C) nucleoplasm (N) and chromatin (Chr) WT extracts showing the subcellular localization of DUXAP8. WT myoblasts were transfected with a plasmid for the overexpression of DUXAP8 and collected after 96 h. GAPDH mRNA and pre-mRNA (pre-GAPDH) were used as cytoplasm, nucleoplasm and chromatin controls. Lane (−) indicates the negative control. Representative results are shown (n = 3).

Source data are available online for this figure.

© 2020 The Authors

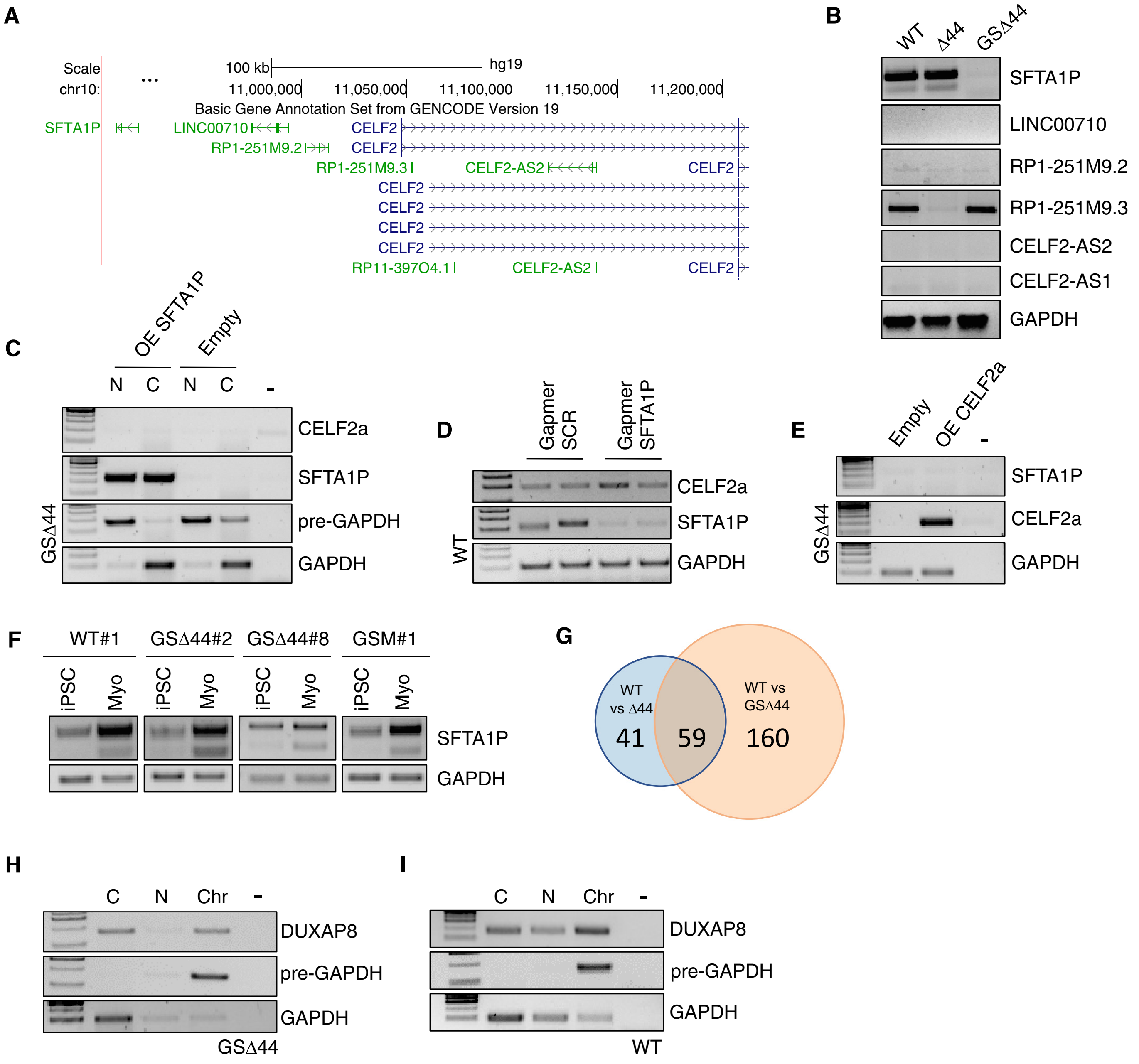

Figure EV4.

© 2020 The Authors
