# Supplementary material for: Trans‐generational epigenetic regulation associated with the amelioration of Duchenne Muscular Dystrophy
Source: EMBO Mol Med. 2020 Jun 29;12(8):e12063. doi: 10.15252/emmm.202012063 (PMC7411655; doi:10.15252/emmm.202012063)

Figure EV4B

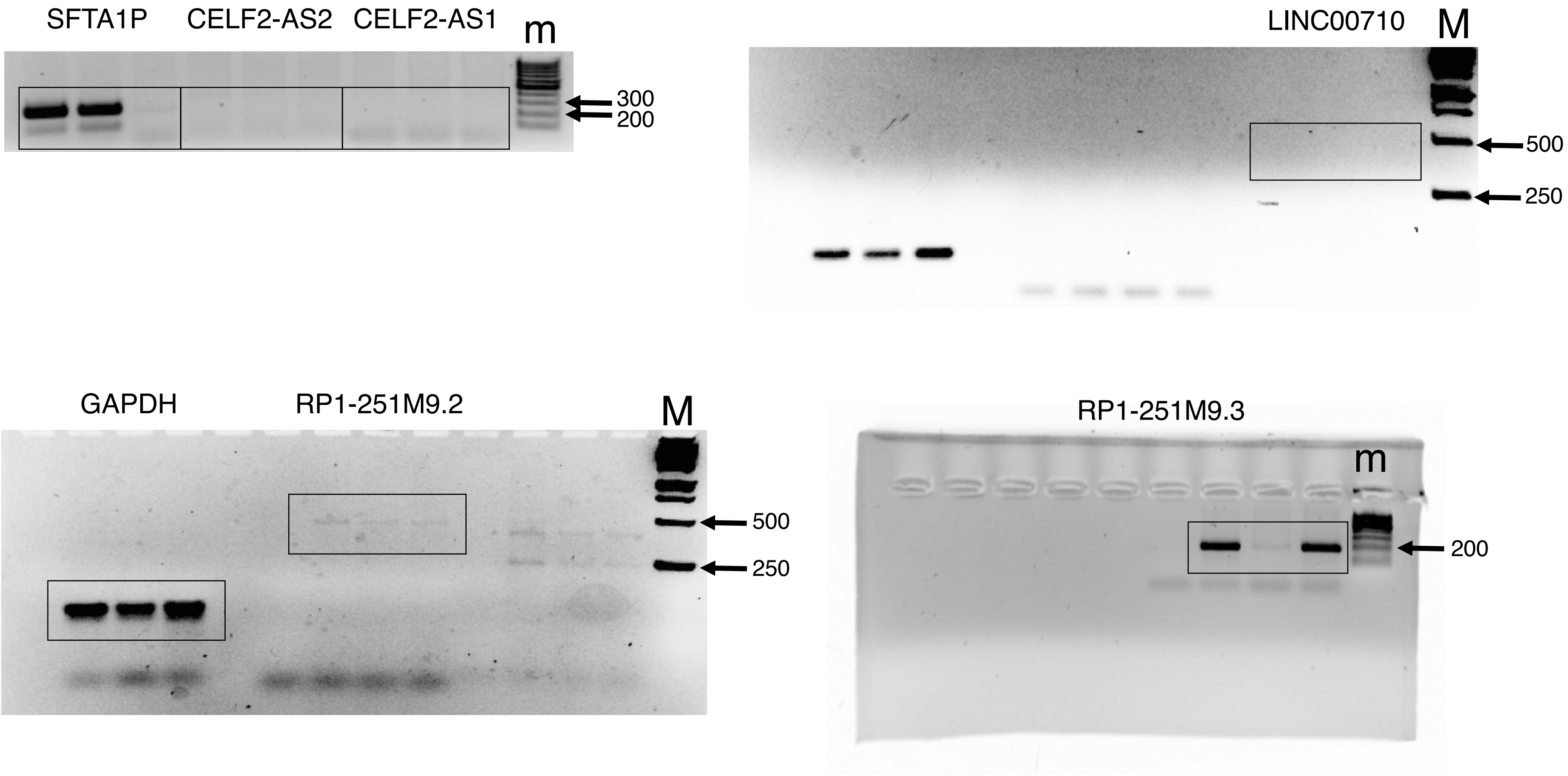

m= 100bp marker
M=1kb marker

Figure EV4C

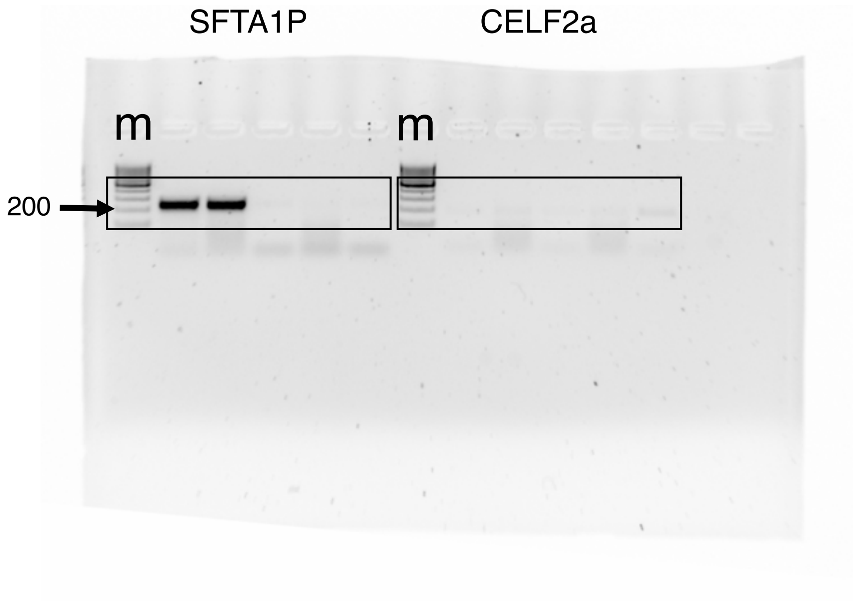

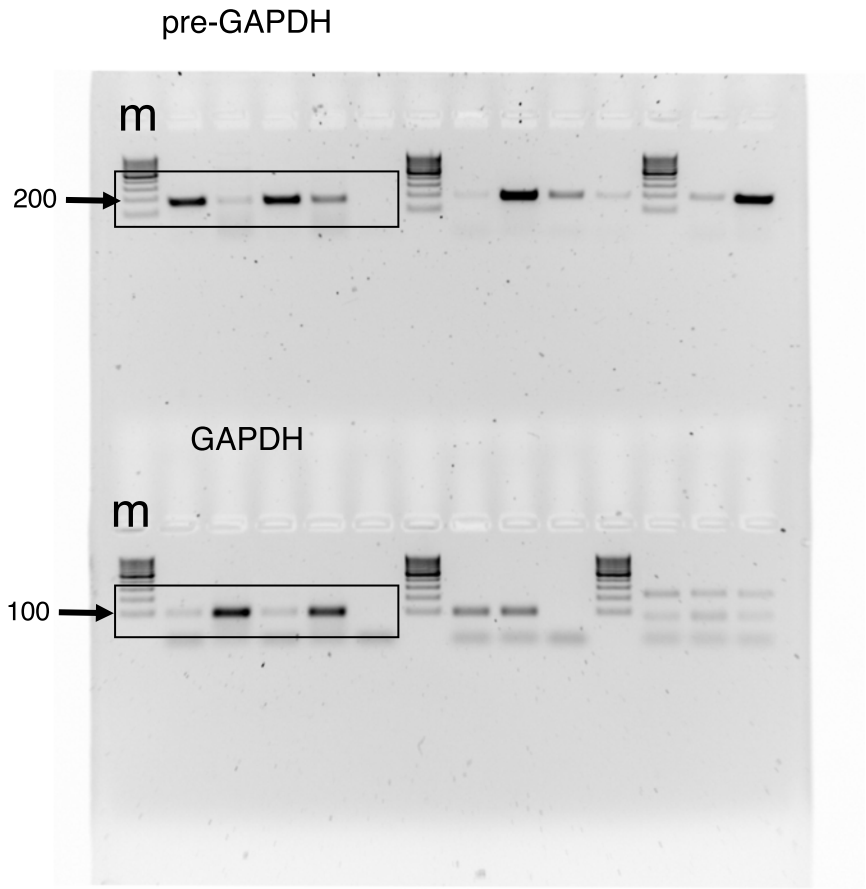

Figure EV4D

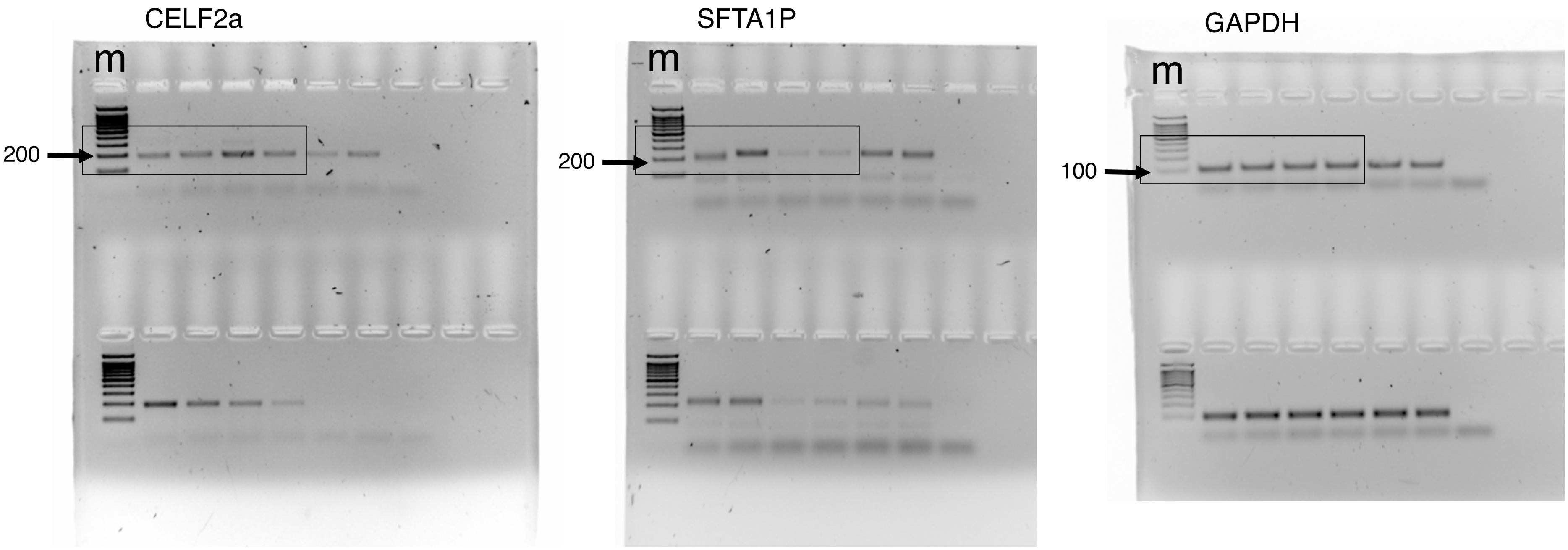

Figure EV4E

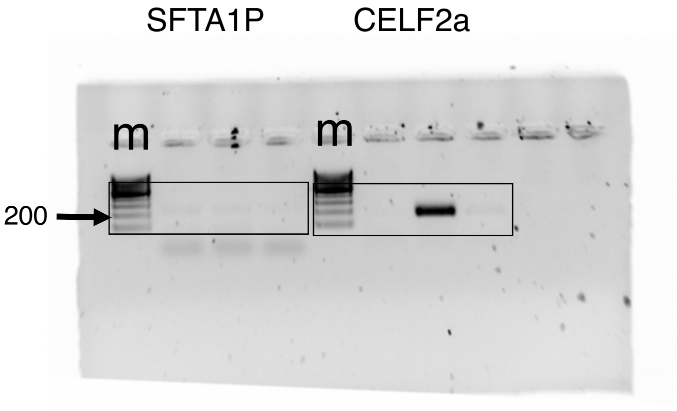

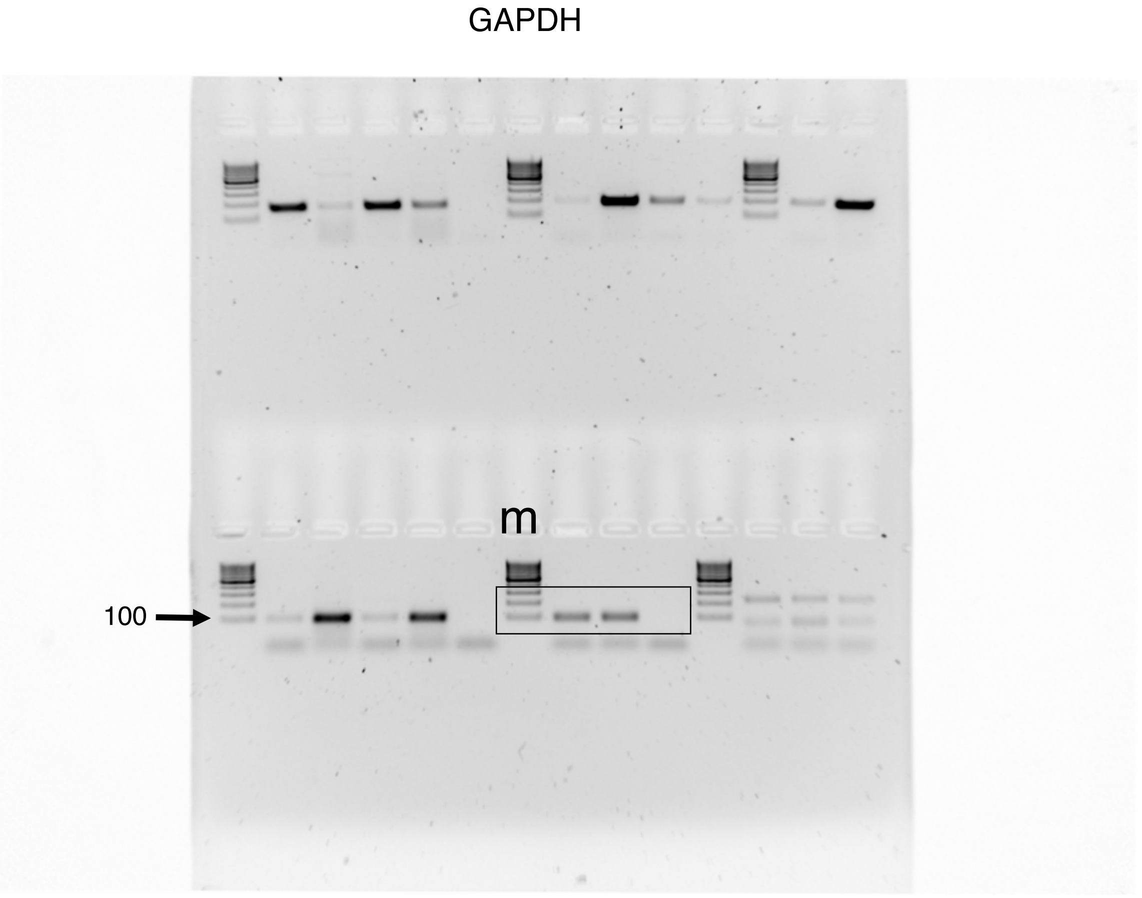

Figure EV4F

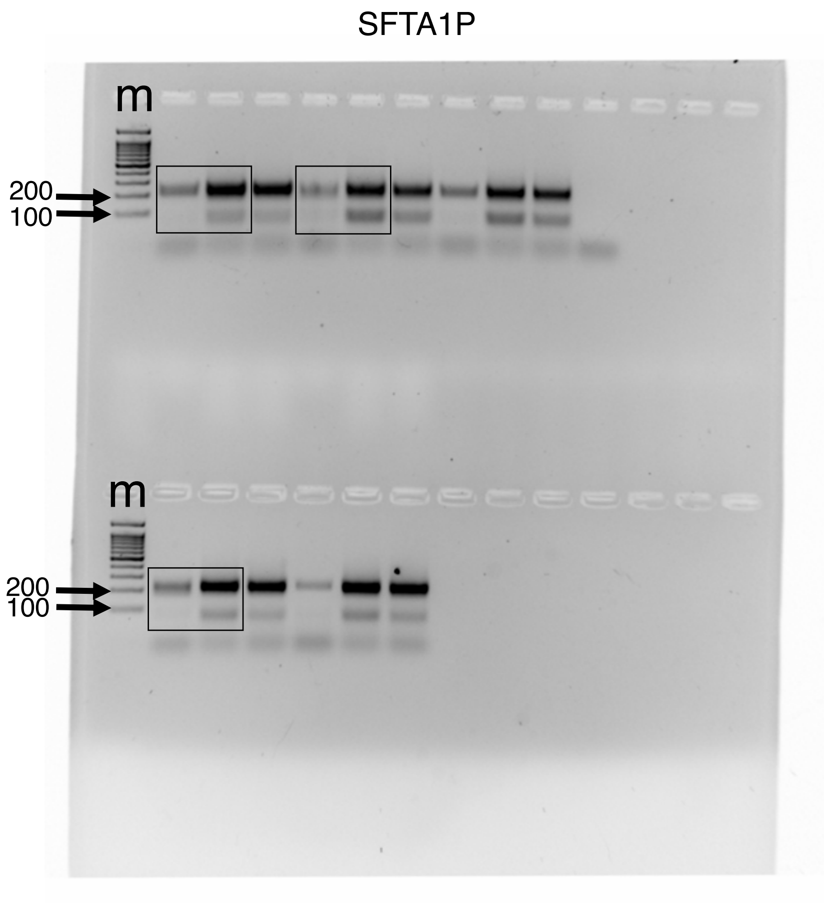

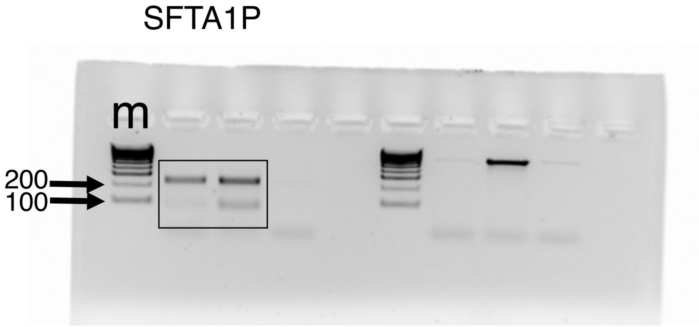

Figure EV4H

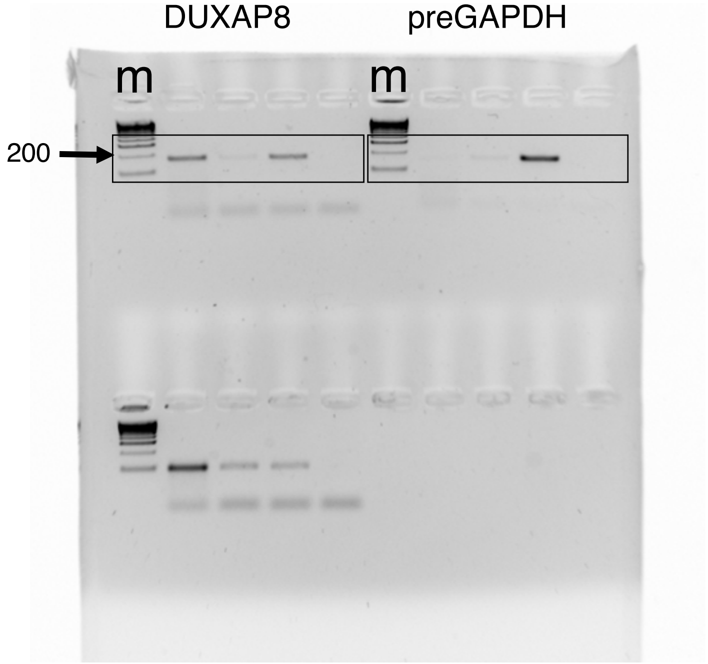

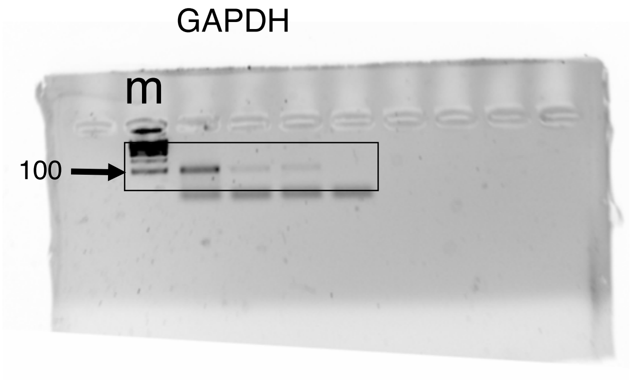

Figure EV4I

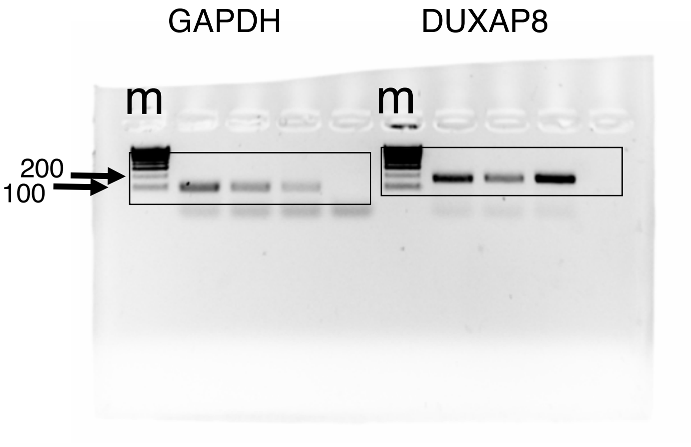

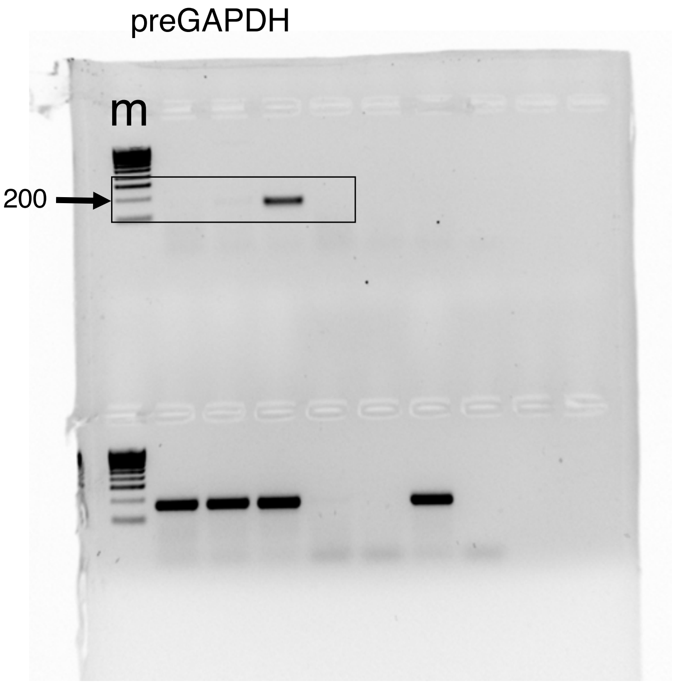

Supplement: Supplementary file 3 — Source Data for Expanded View [file EMMM-12-e12063-s006.zip › Source_Data_FigEV4.pdf]
