# Supplementary figures and images for: Trans‐generational epigenetic regulation associated with the amelioration of Duchenne Muscular Dystrophy
Source: EMBO Mol Med. 2020 Jun 29;12(8):e12063. doi: 10.15252/emmm.202012063 (PMC7411655; doi:10.15252/emmm.202012063)

Figure EV2C

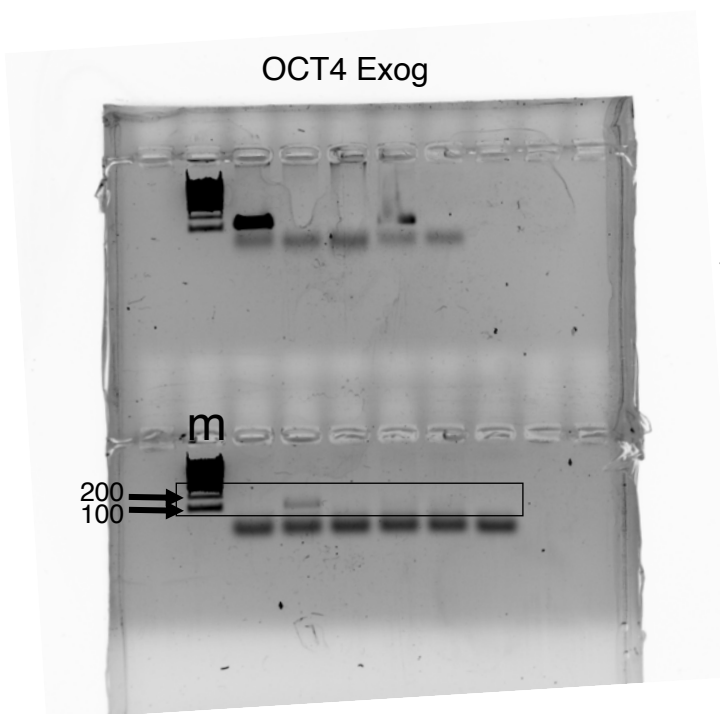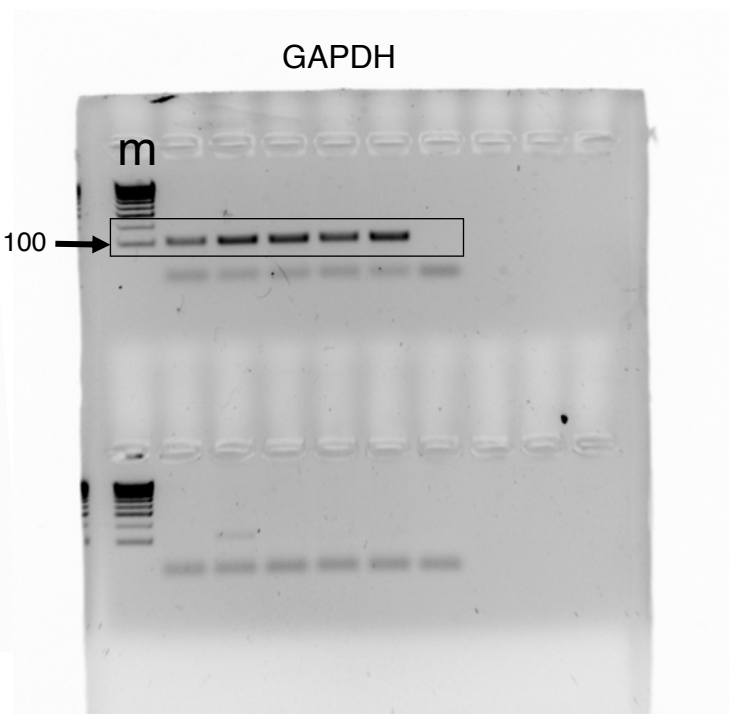

m= 100bp marker  
M=1kb marker

Figure EV2E (left)

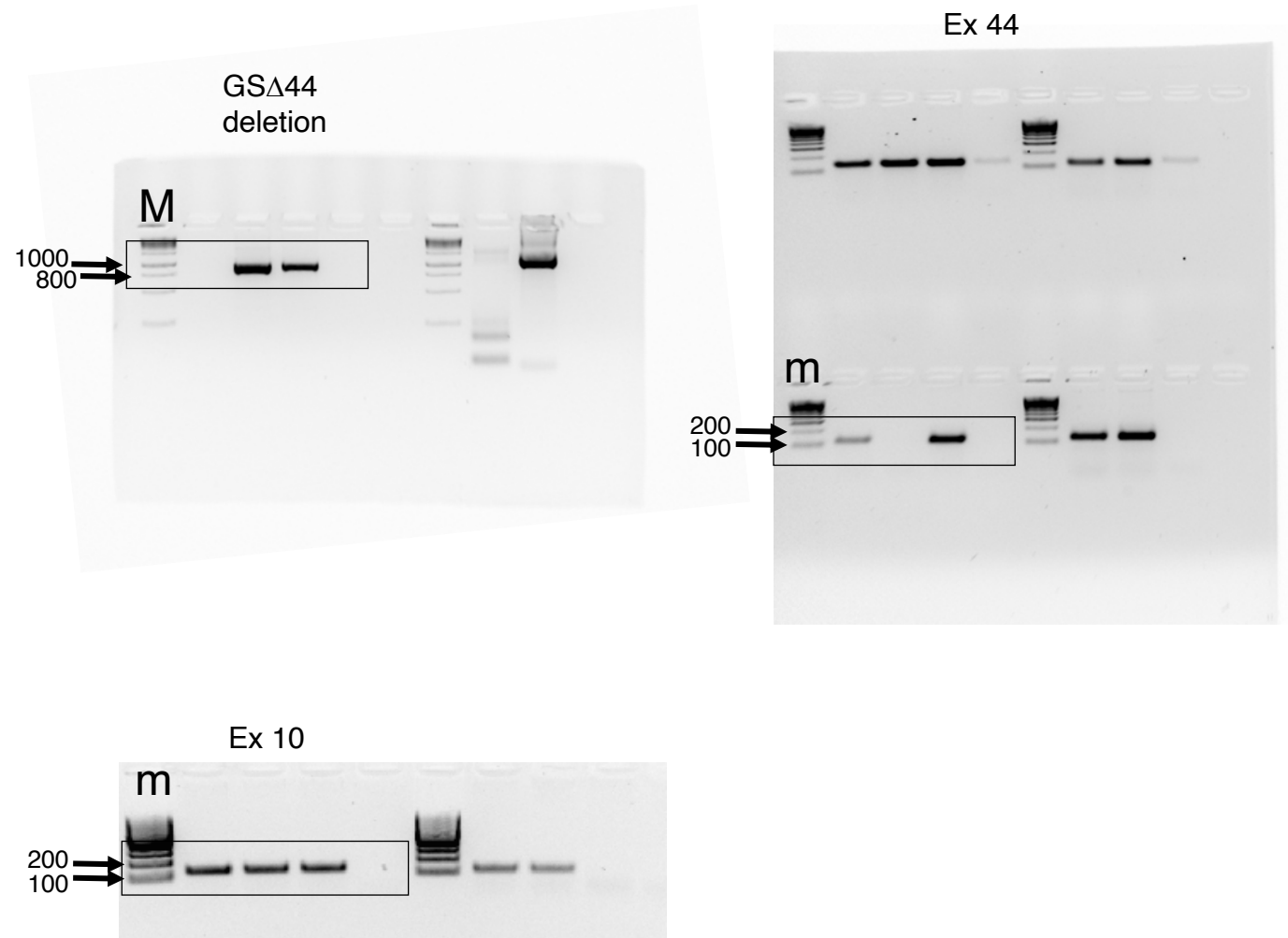

Figure EV2E (right)

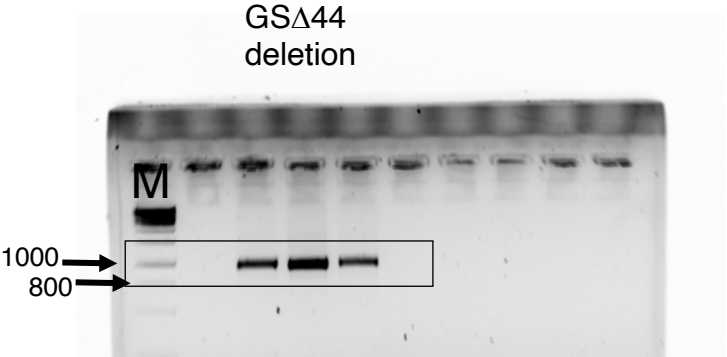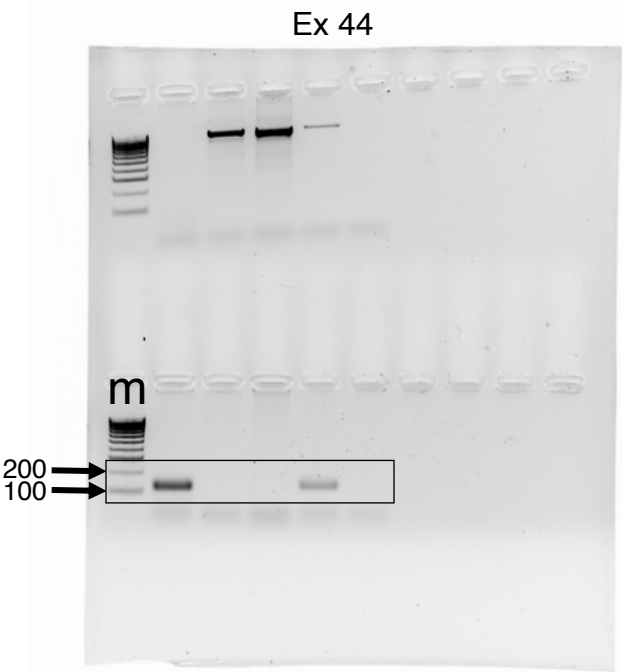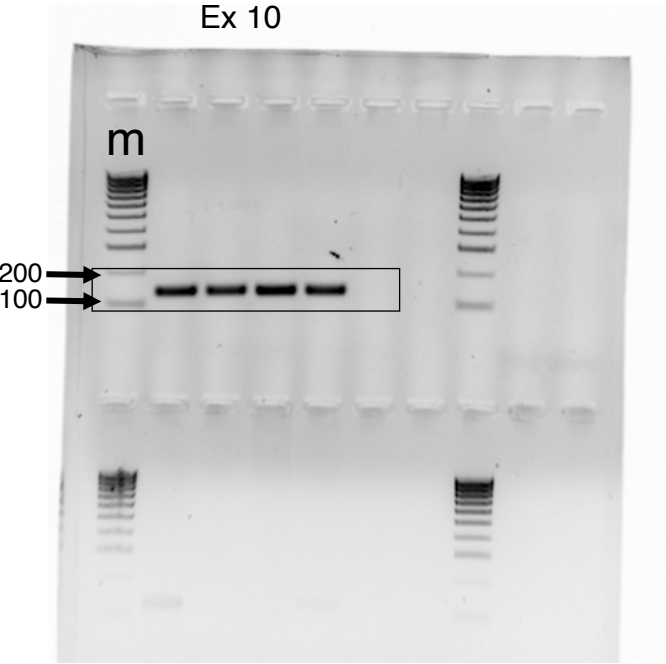

Figure EV2G

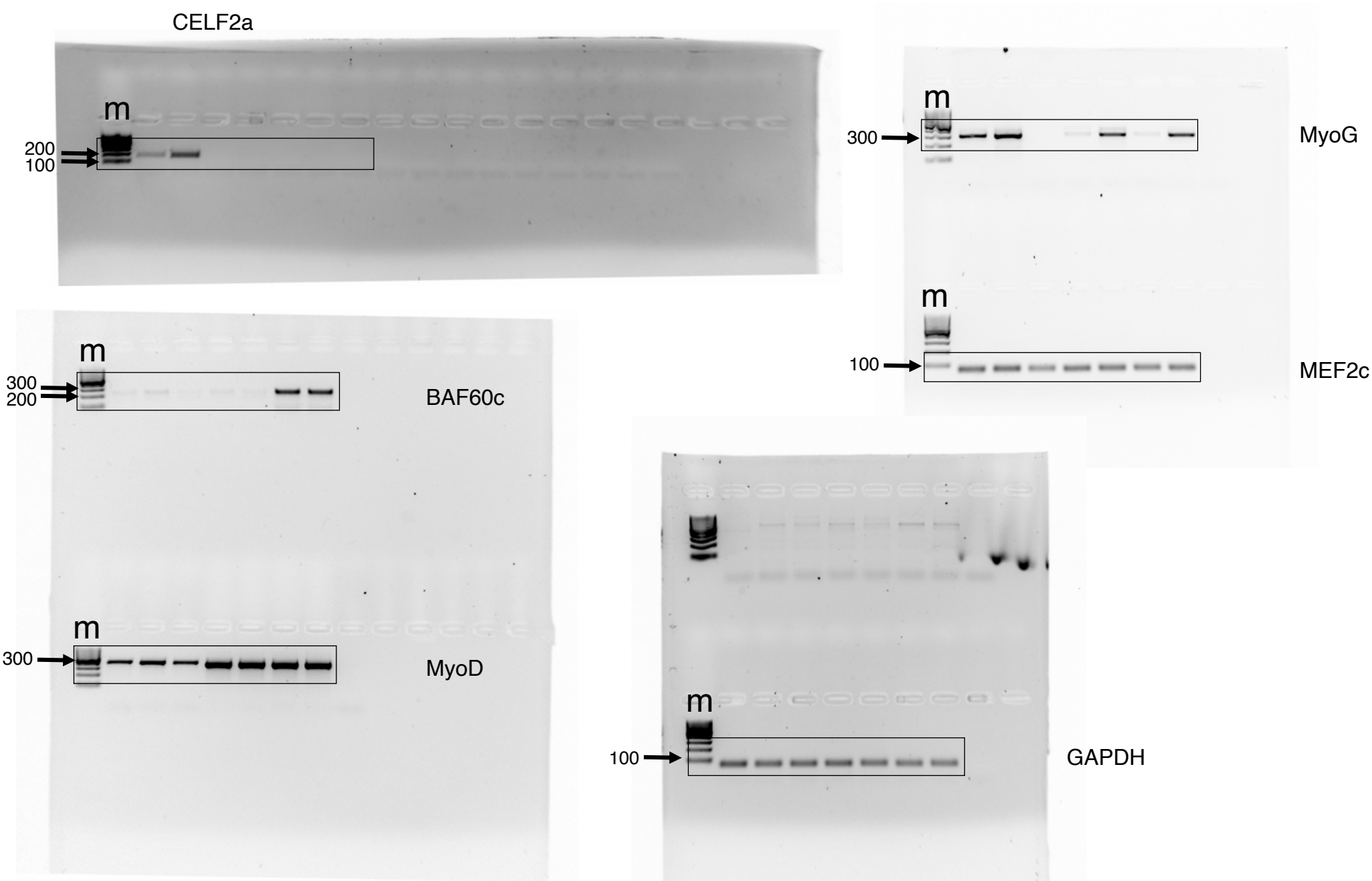

Figure EV2H

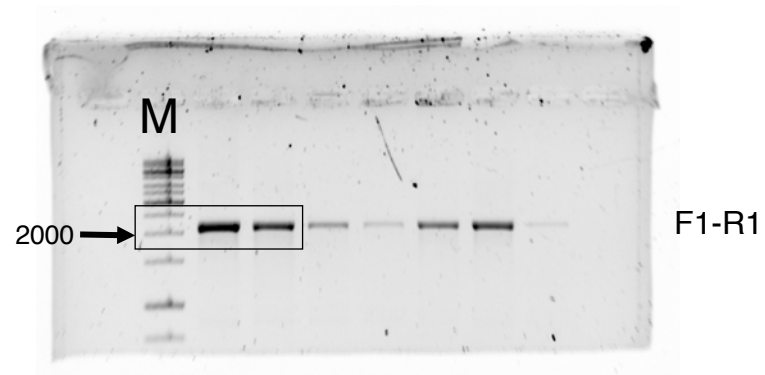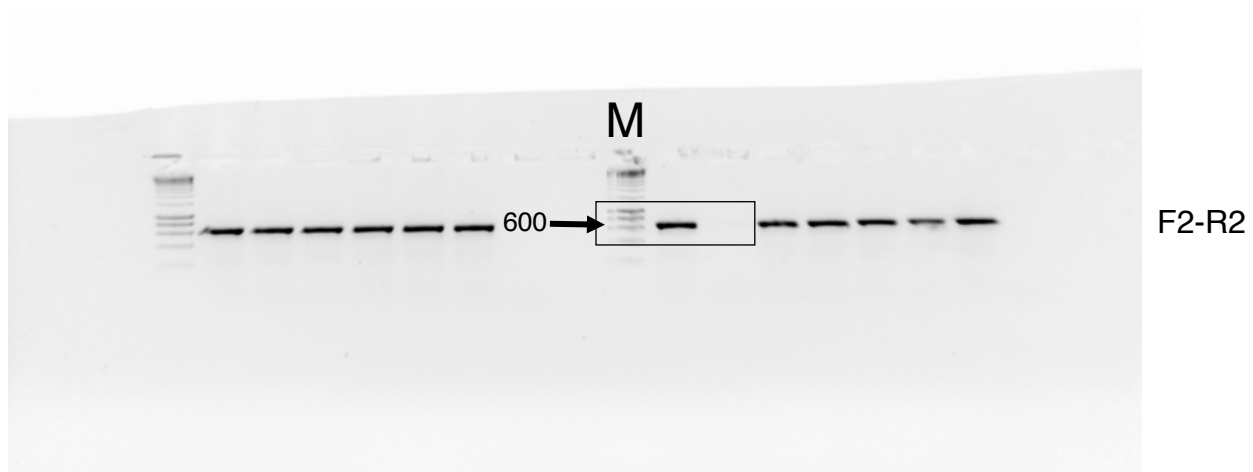

Supplement: Supplementary file 3 — Source Data for Expanded View [file EMMM-12-e12063-s006.zip › Source_Data_FigEV2.pdf]

Figure EV1C

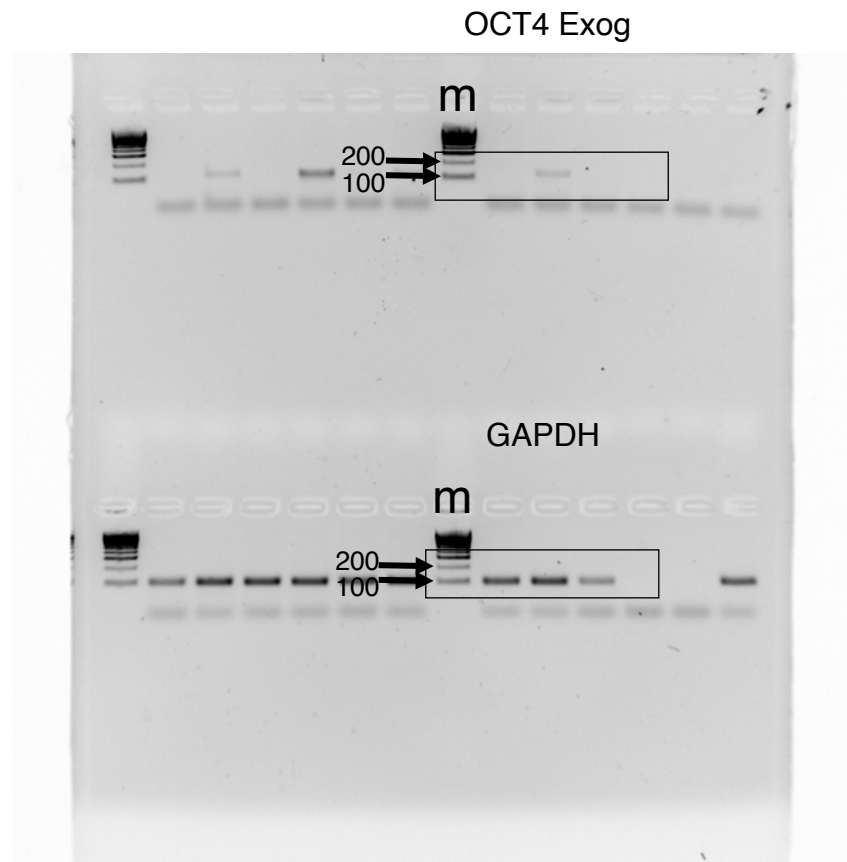

m= 100bp marker  
M=1kb marker

Figure EV1E (left)

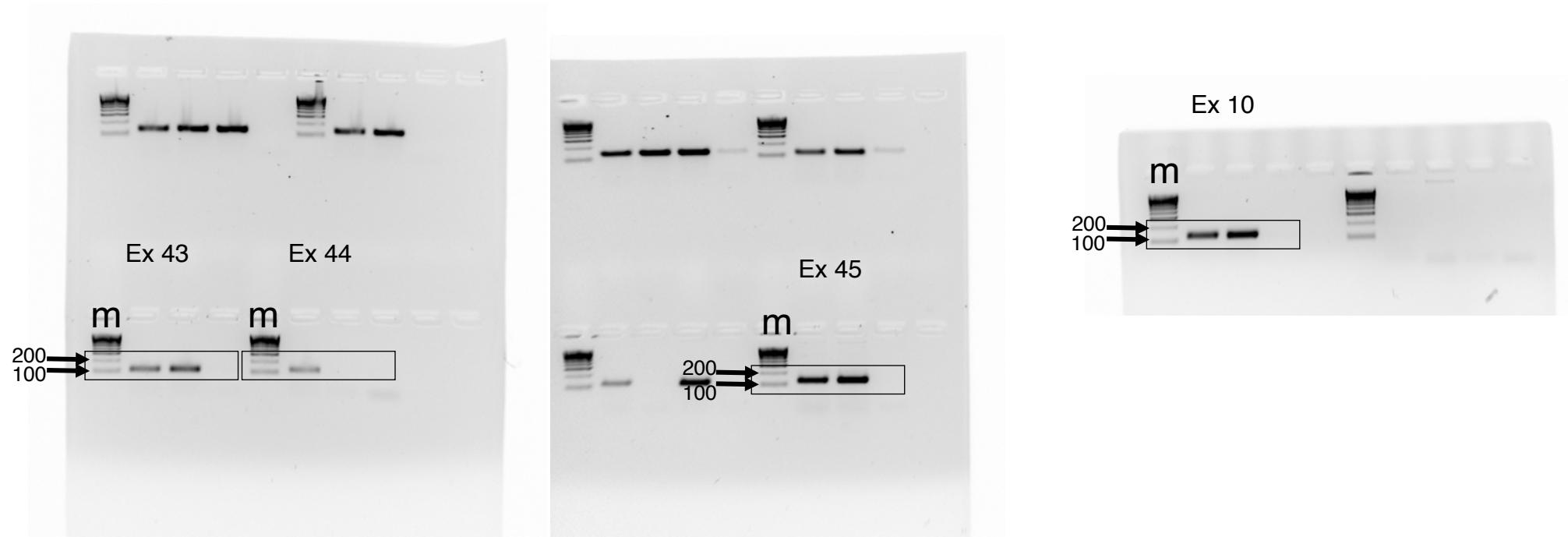

Figure EV1E (right)

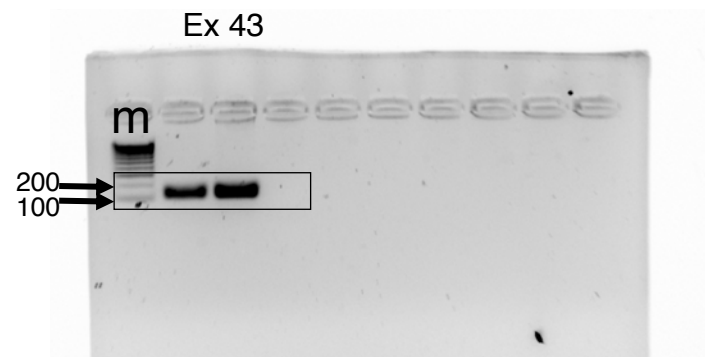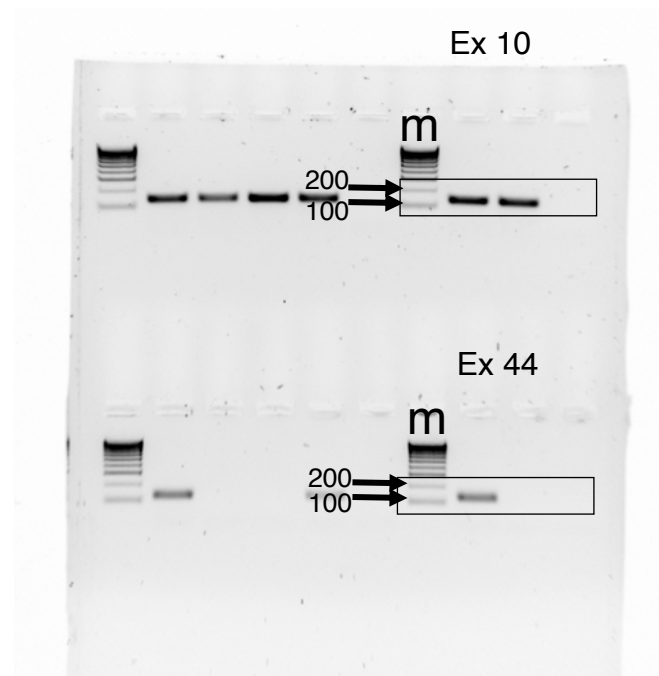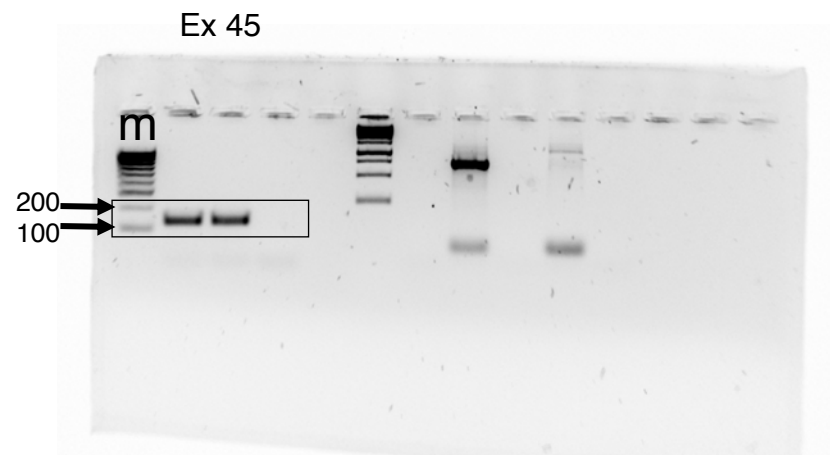

Figure EV1F

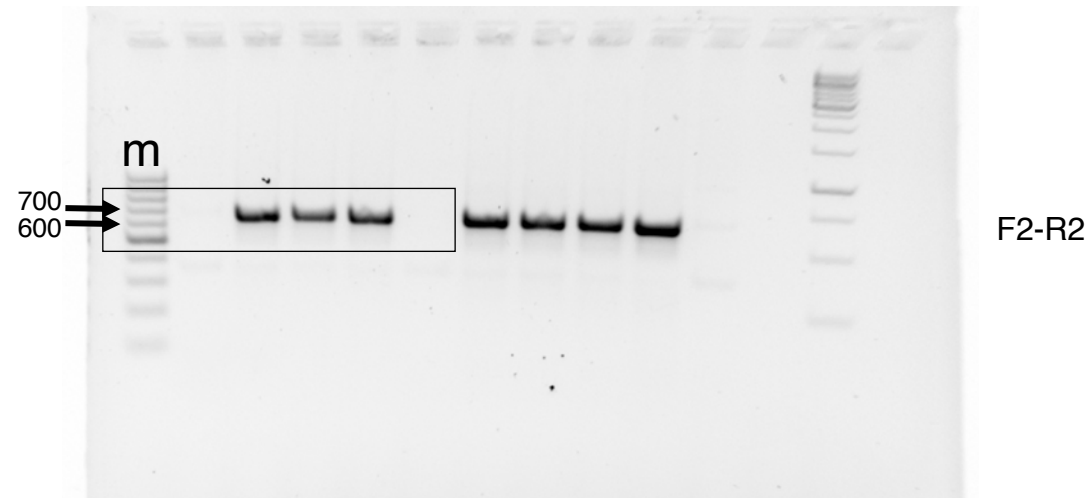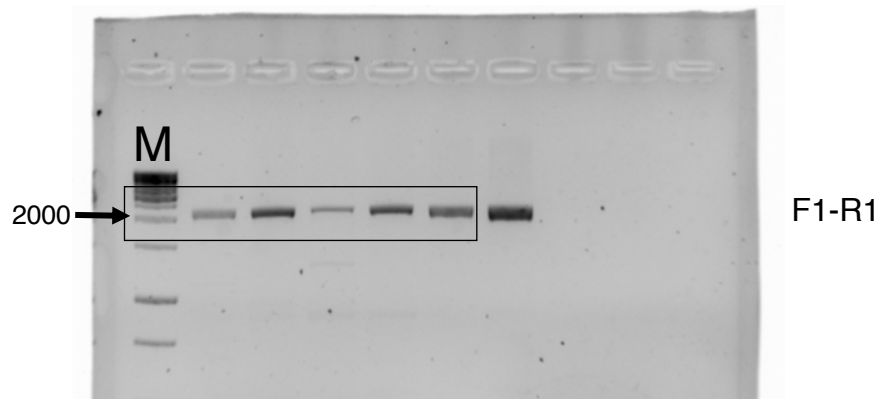

Supplement: Supplementary file 3 — Source Data for Expanded View [file EMMM-12-e12063-s006.zip › Source_Data_FigEV1.pdf]

Figure 1A

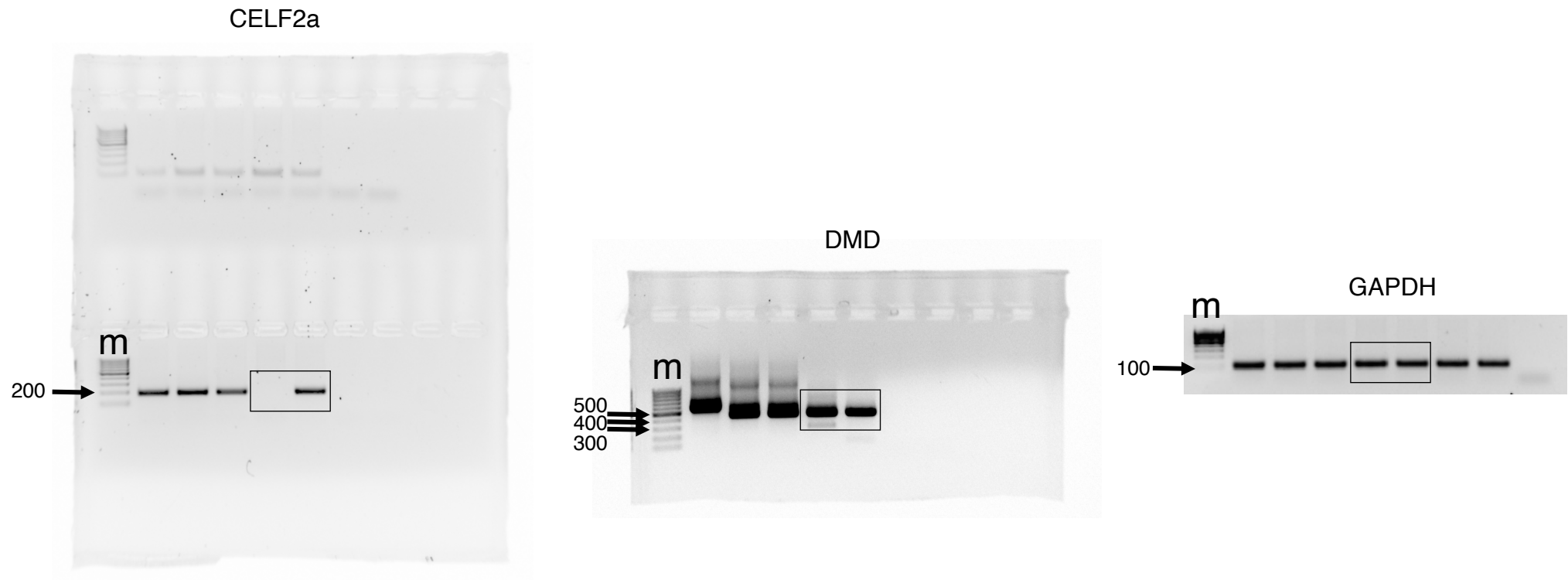

m= 100bp marker  
M=1kb marker

Figure 1B

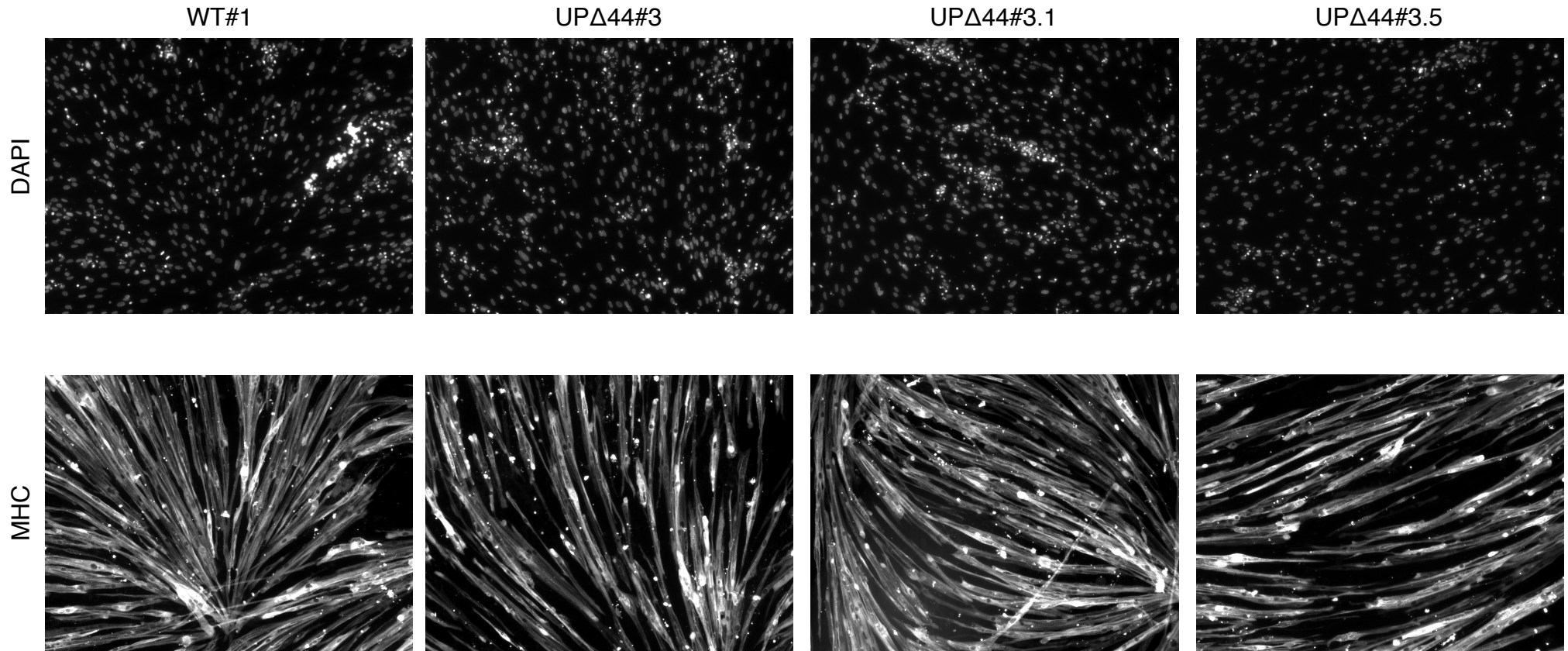

Figure 1D

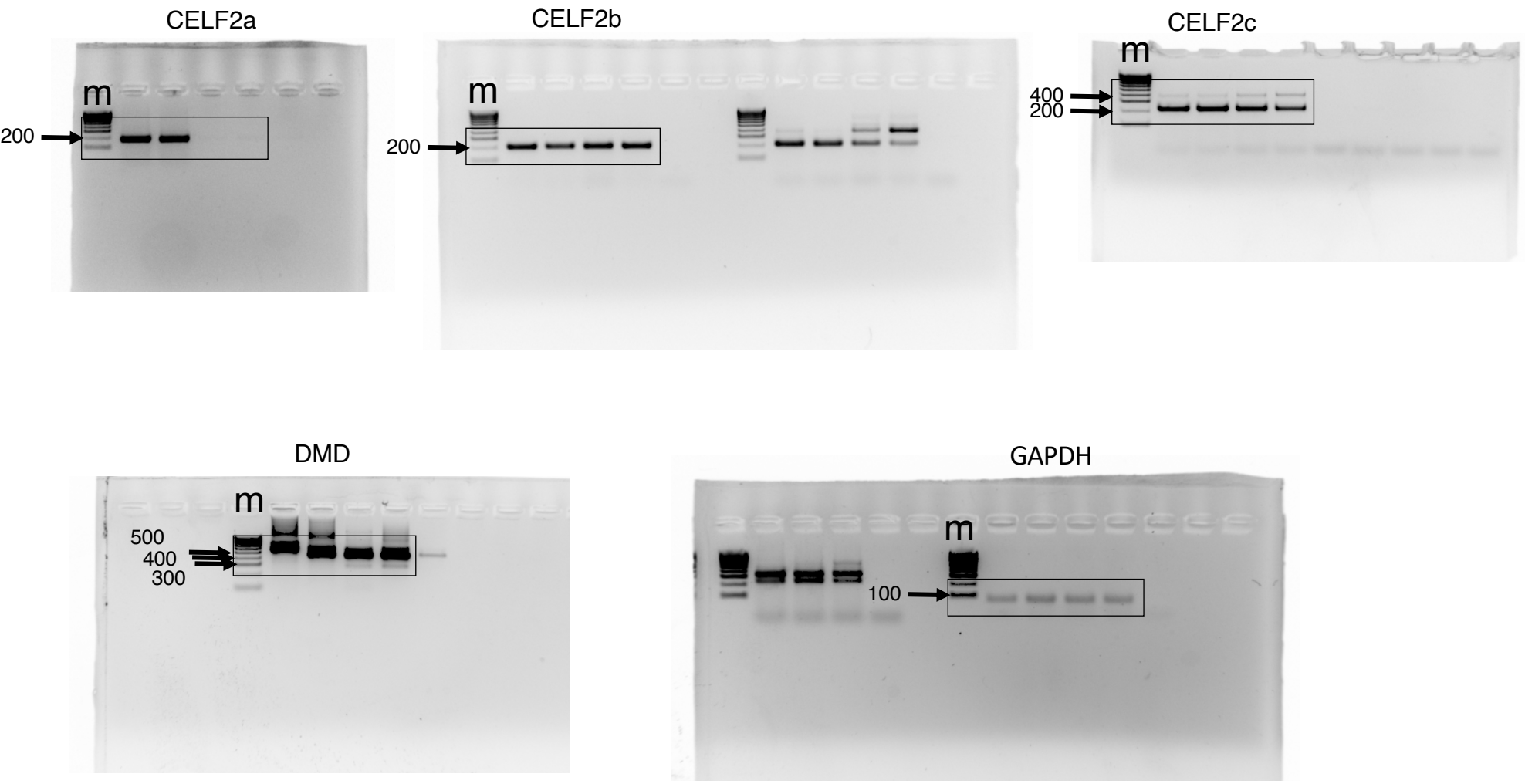

Figure 1E

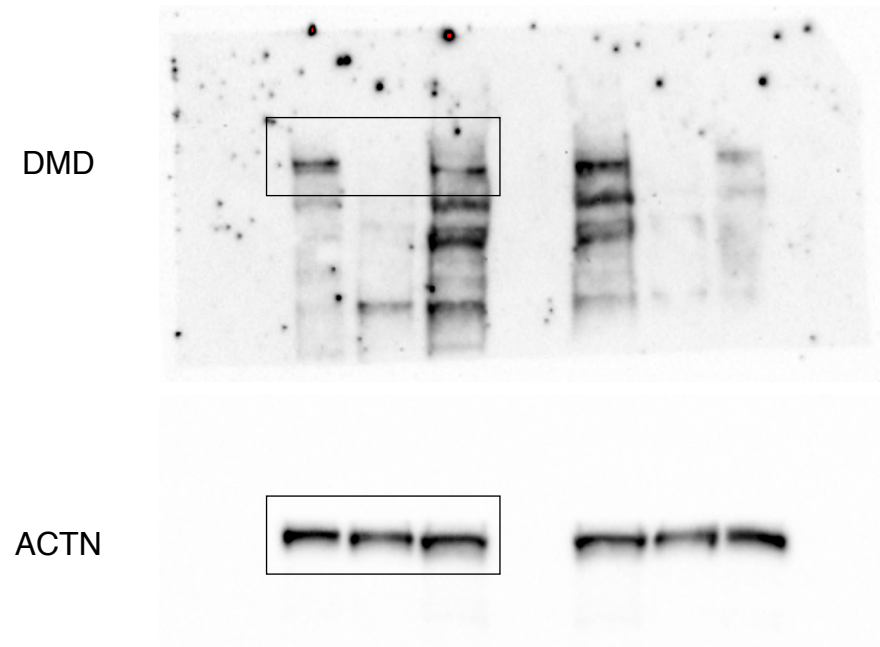

Supplement: Supplementary file 5 — Source Data for Figure 1 [file EMMM-12-e12063-s003.pdf]

Figure 4B

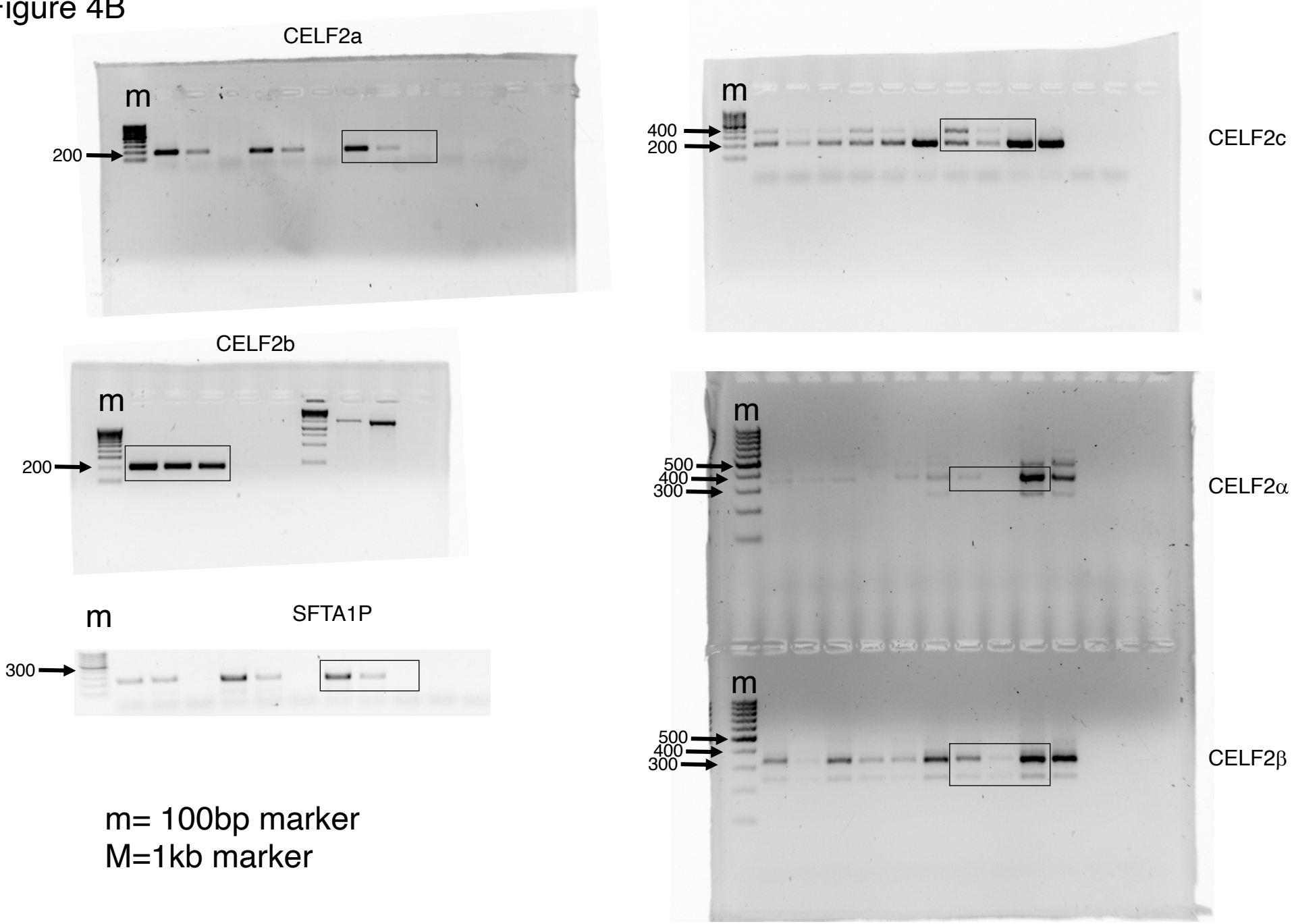

Figure 4B

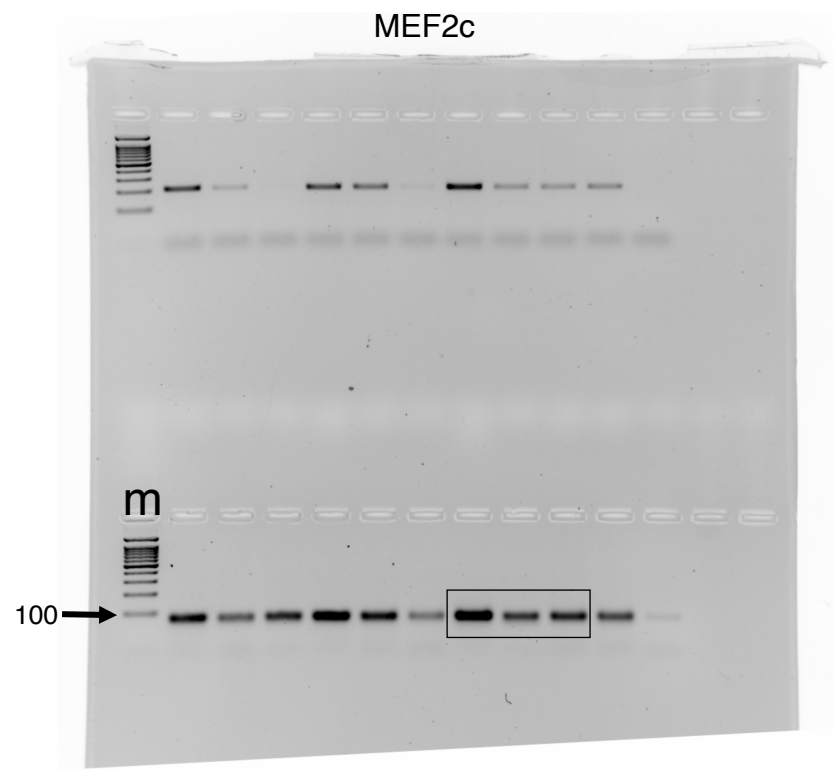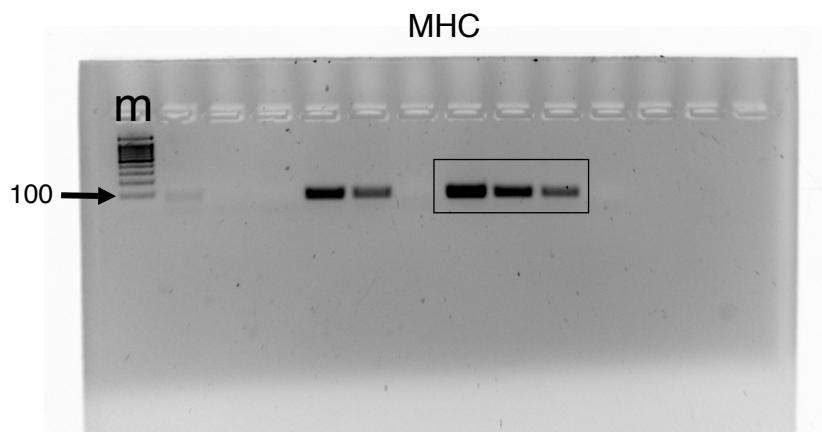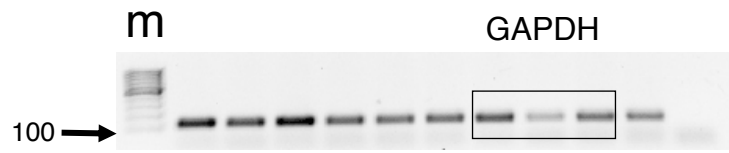

Figure 4C

CELF2a

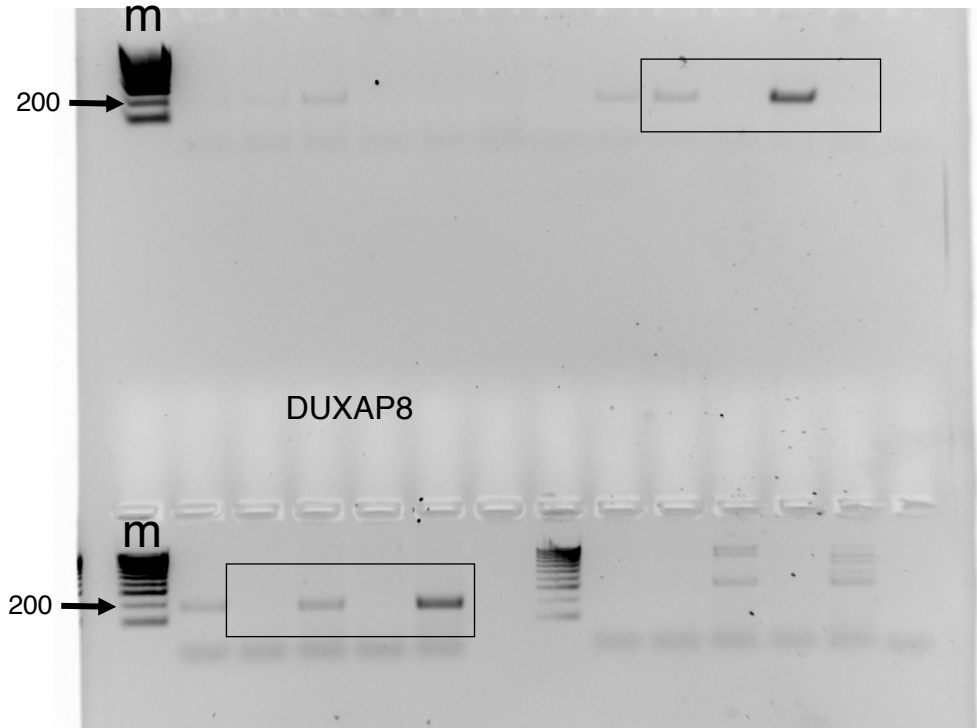

GAPDH

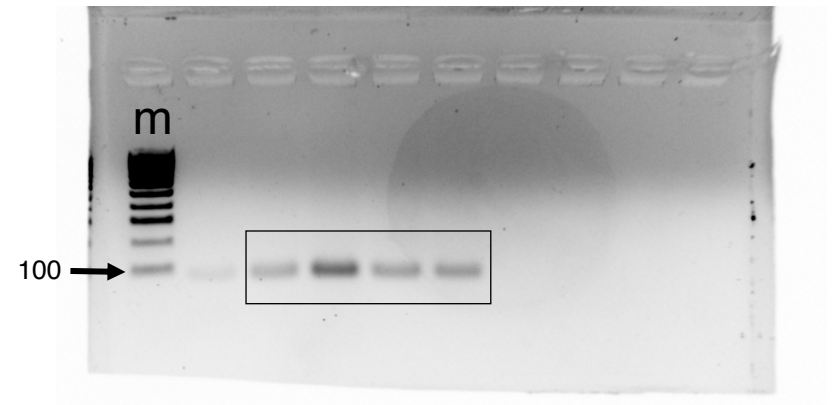

Figure 4D

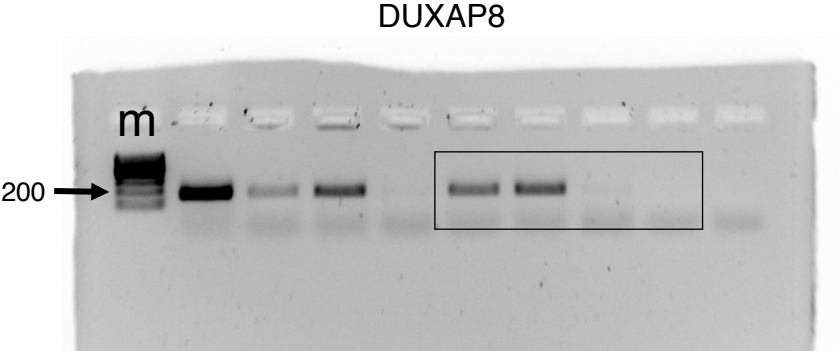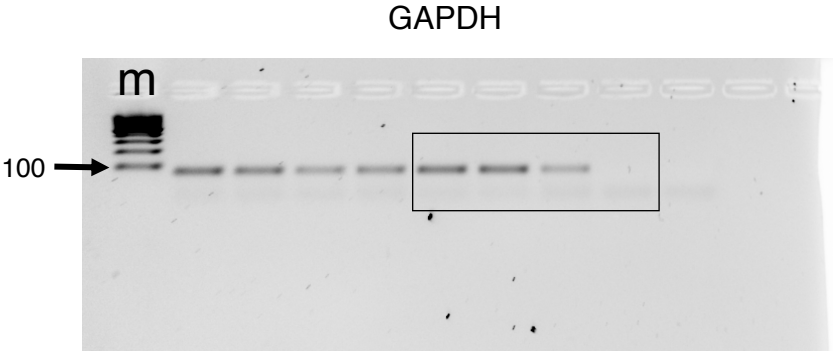

## Figure 4E

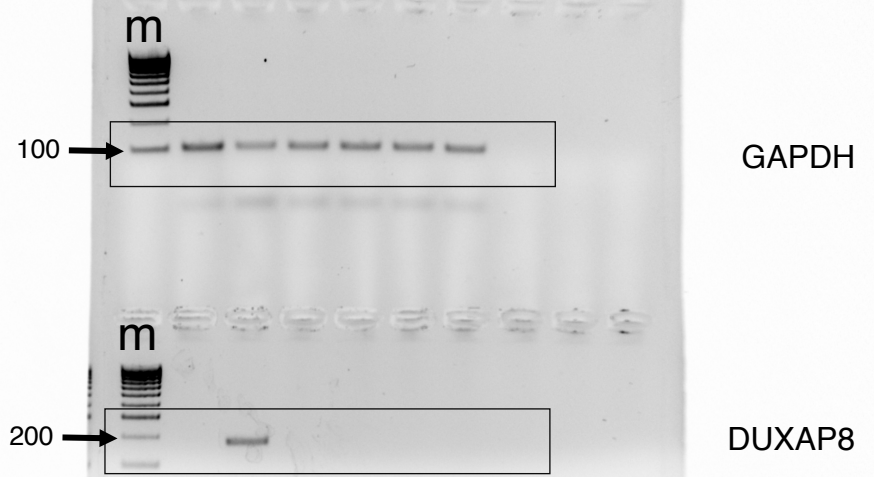

Figure 4F

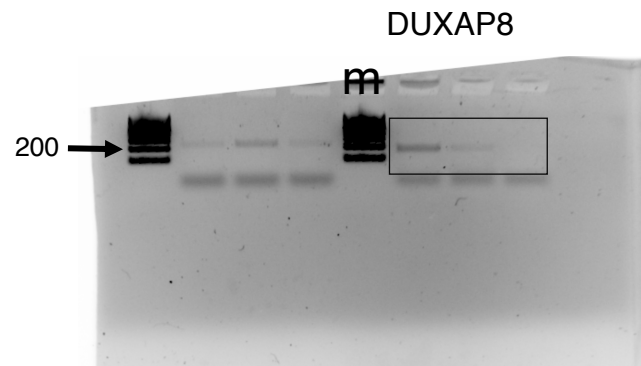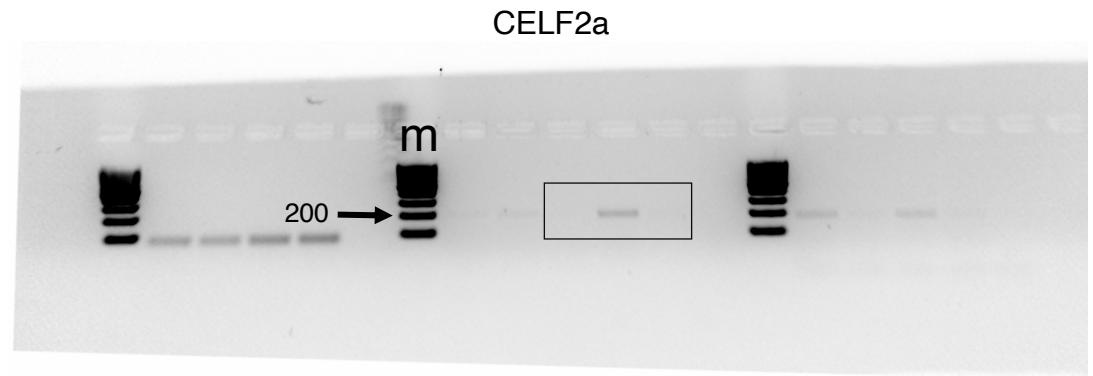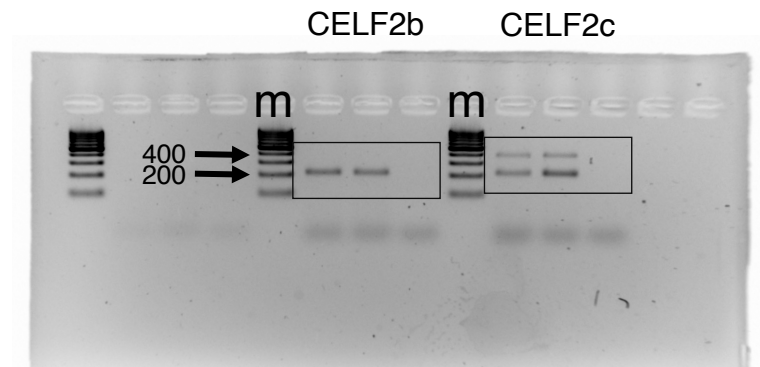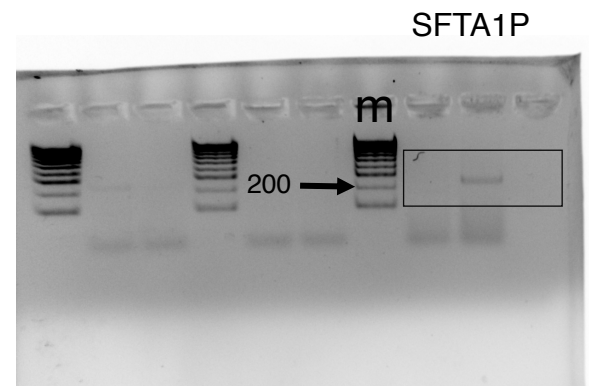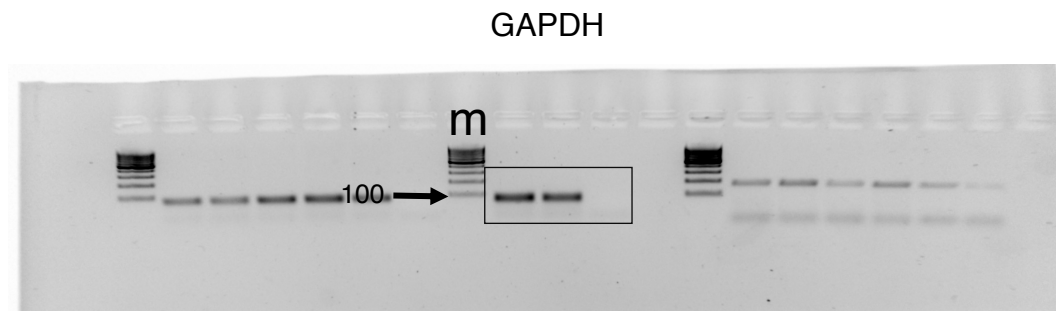

Supplement: Supplementary file 7 — Source Data for Figure 4 [file EMMM-12-e12063-s005.pdf]
