# Supplementary material for: Trans‐generational epigenetic regulation associated with the amelioration of Duchenne Muscular Dystrophy
Source: EMBO Mol Med. 2020 Jun 29;12(8):e12063. doi: 10.15252/emmm.202012063 (PMC7411655; doi:10.15252/emmm.202012063)

Figure 2A

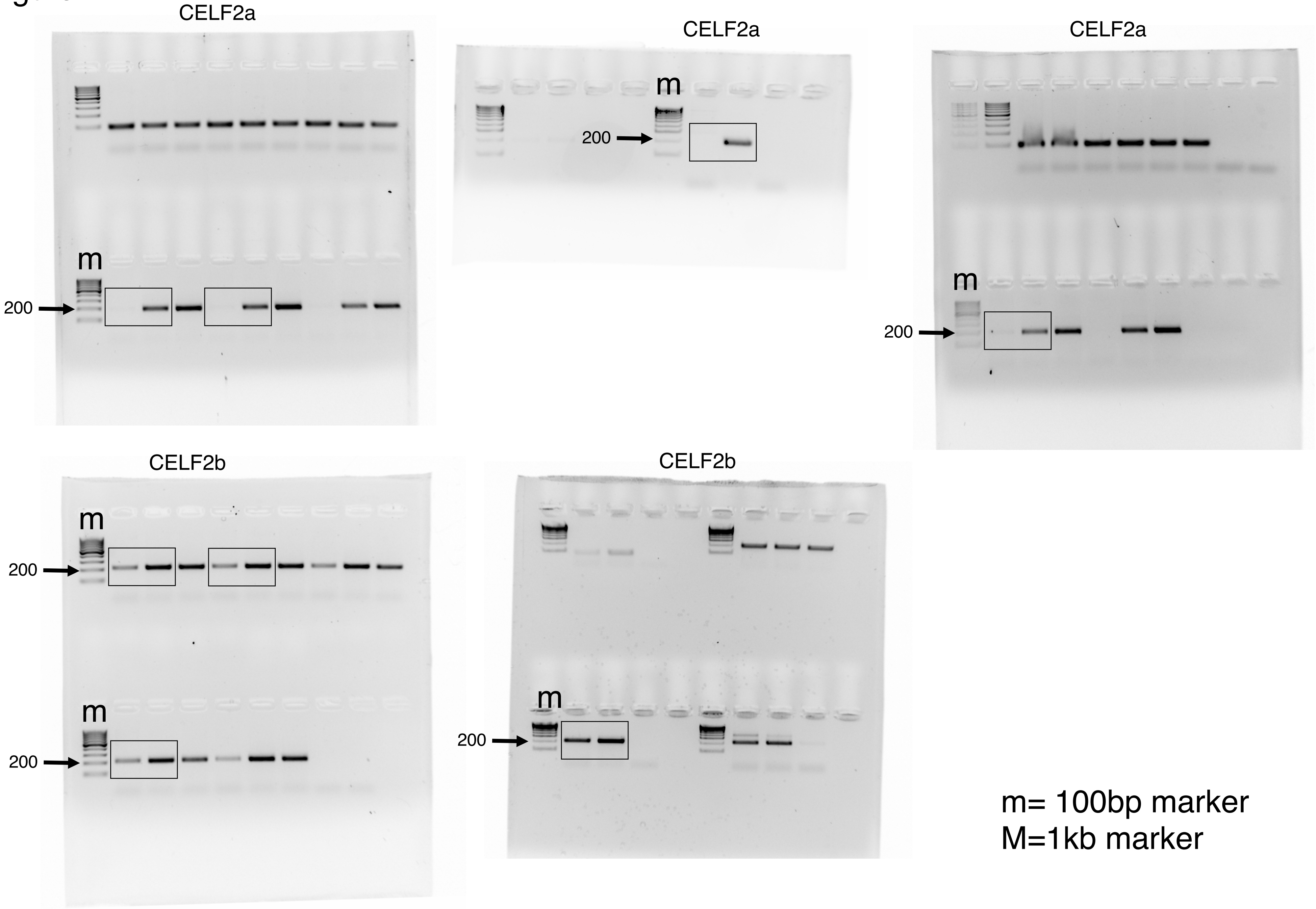
CELF2a
m
200
CELF2a
m
200
CELF2a
m
200
CELF2b
m
200
m
200
CELF2b
m
200
m= 100bp marker
M=1kb marker

Figure 2A

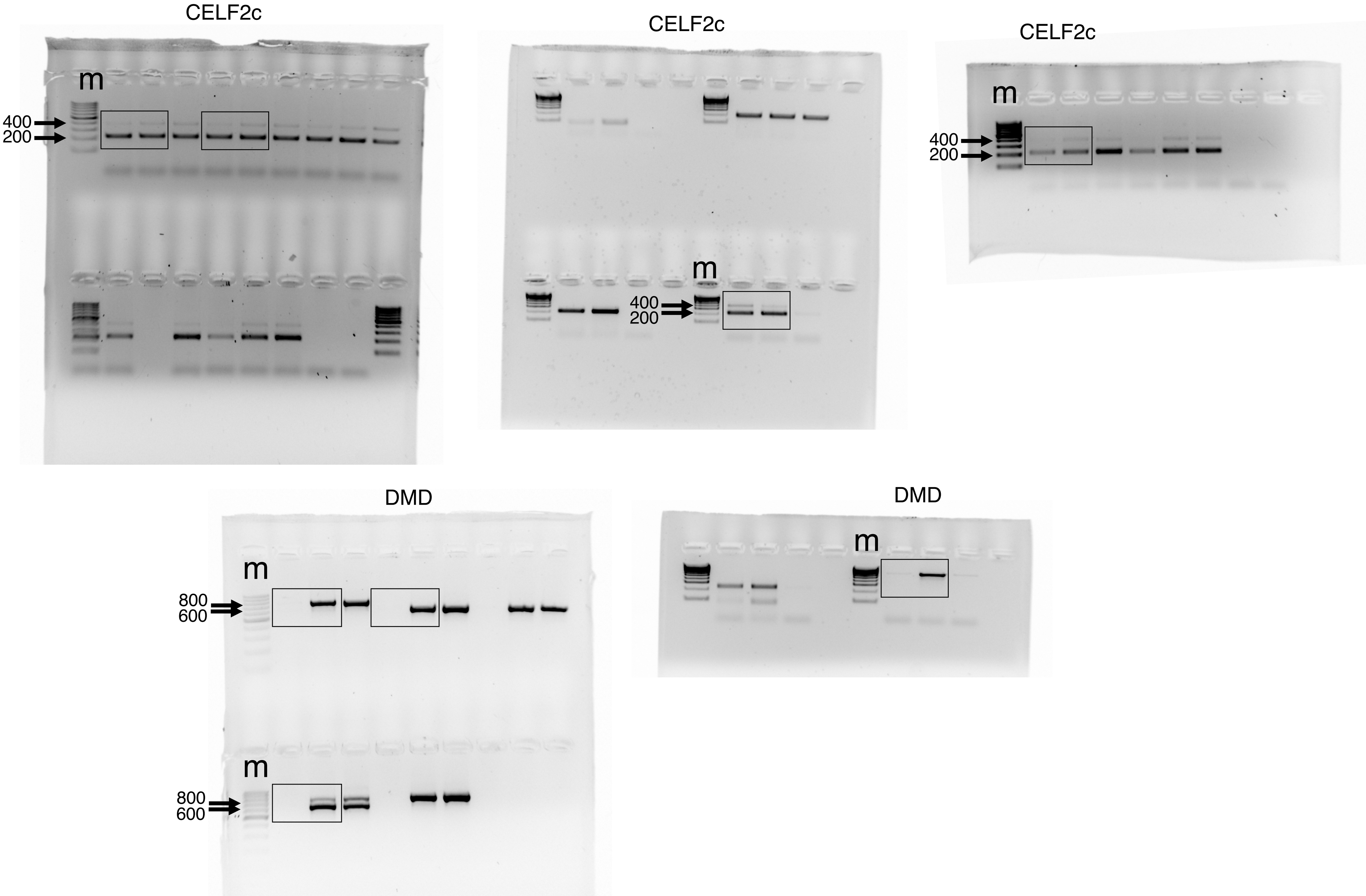
CELF2c
m
400
200
CELF2c
m
400
200
CELF2c
m
400
200
DMD
m
800
600
m
800
600
DMD
m

Figure 2A

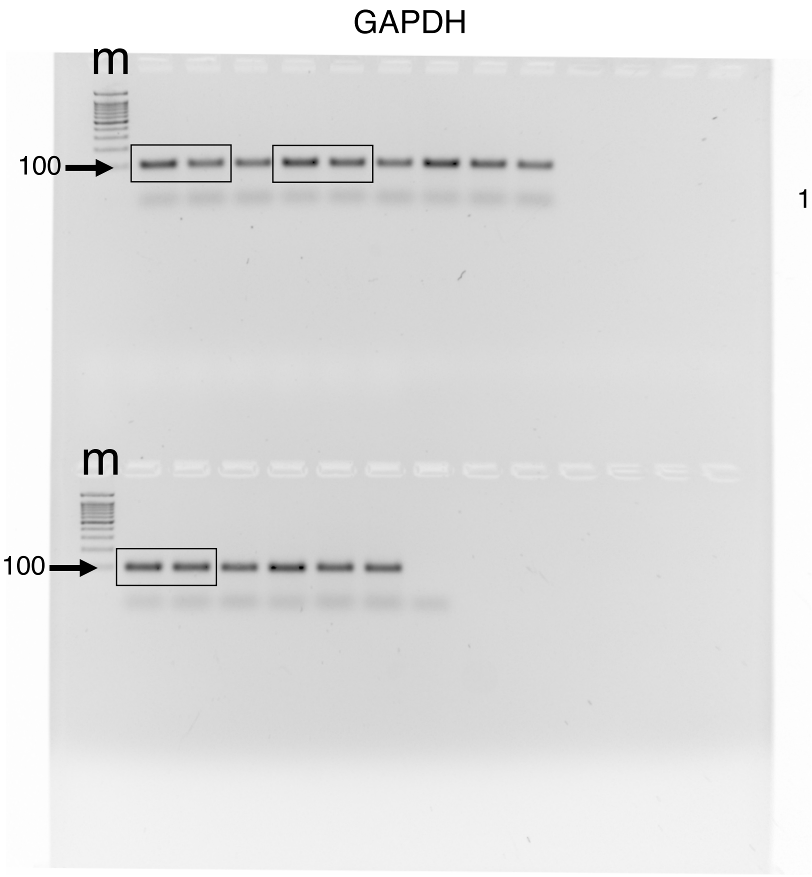

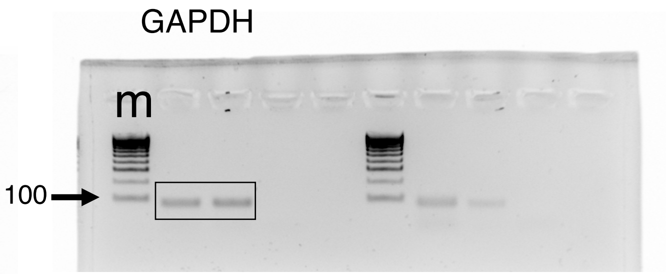

Figure 2B

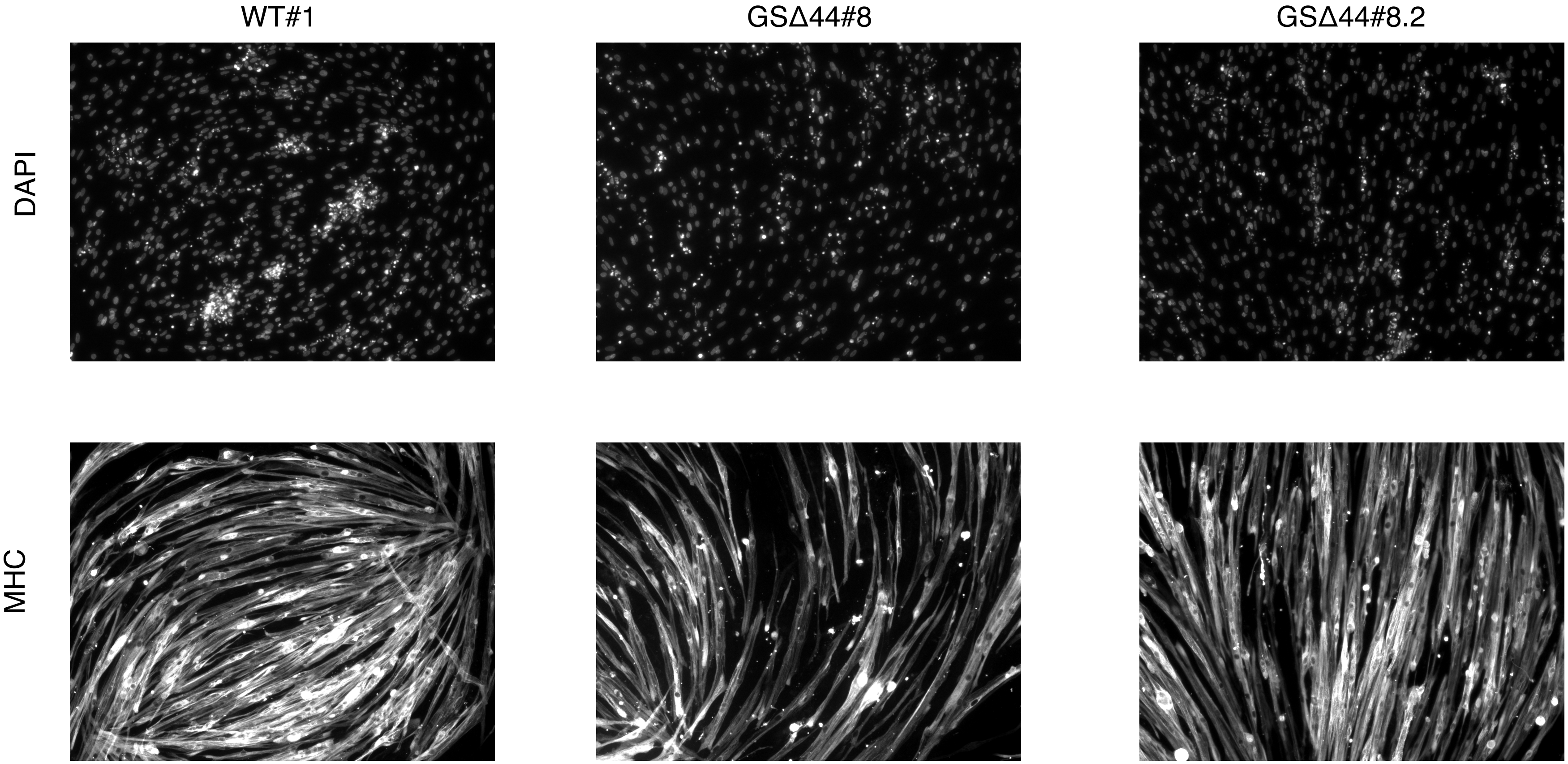

Figure 2D

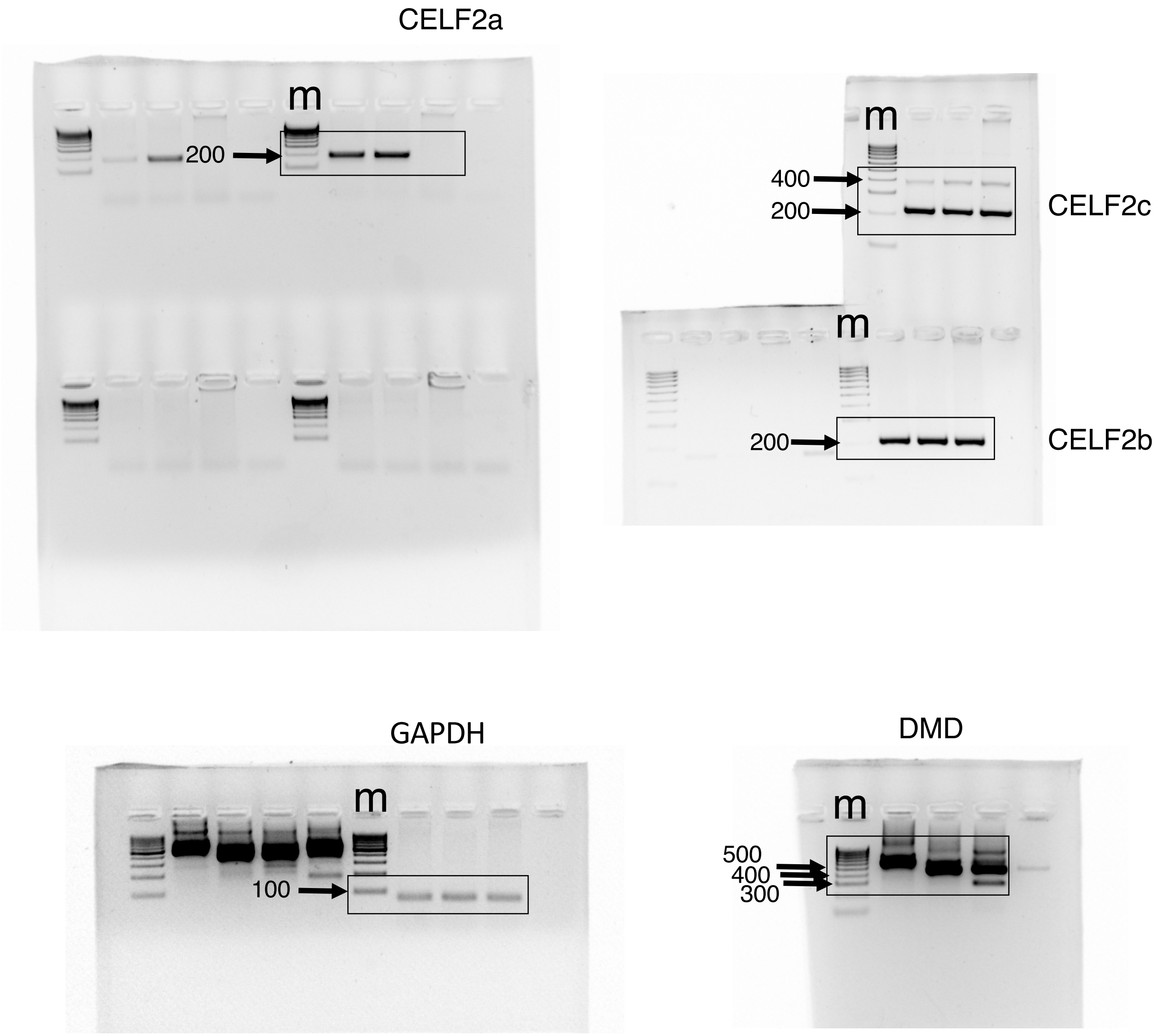
CELF2a
m
200
m
400
200
CELF2c
m
200
CELF2b
GAPDH
m
100
DMD
m
500
400
300

Figure 2E

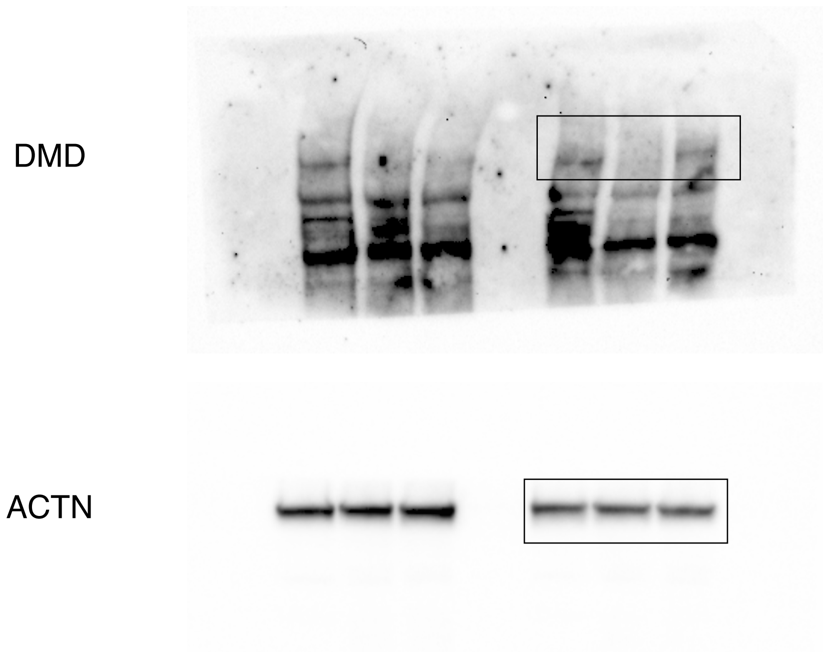

Supplement: Supplementary file 6 — Source Data for Figure 2 [file EMMM-12-e12063-s004.pdf]
